# TFEB-mediated lysosomal exocytosis alleviates high-fat diet–induced lipotoxicity in the kidney

**DOI:** 10.1172/jci.insight.162498

**Published:** 2023-02-22

**Authors:** Jun Nakamura, Takeshi Yamamoto, Yoshitsugu Takabatake, Tomoko Namba-Hamano, Satoshi Minami, Atsushi Takahashi, Jun Matsuda, Shinsuke Sakai, Hiroaki Yonishi, Shihomi Maeda, Sho Matsui, Isao Matsui, Takayuki Hamano, Masatomo Takahashi, Maiko Goto, Yoshihiro Izumi, Takeshi Bamba, Miwa Sasai, Masahiro Yamamoto, Taiji Matsusaka, Fumio Niimura, Motoko Yanagita, Shuhei Nakamura, Tamotsu Yoshimori, Andrea Ballabio, Yoshitaka Isaka

**Affiliations:** 1 Department of Nephrology, Osaka University Graduate School of Medicine, Osaka, Japan.; 2 Department of Nephrology, Nagoya City University Graduate School of Medical Sciences, Aichi, Japan.; 3 Division of Metabolomics, Medical Institute of Bioregulation, Kyushu University, Fukuoka, Japan.; 4 Department of Immunoparasitology, Research Institute for Microbial Diseases, and; 5 Laboratory of Immunoparasitology, World Premier International Research Center Initiative Immunology Frontier Research Center, Osaka University, Osaka, Japan.; 6 Institute of Medical Sciences and Department of Basic Medical Science, and; 7 Department of Pediatrics, Tokai University School of Medicine, Kanagawa, Japan.; 8 Department of Nephrology, Kyoto University Graduate School of Medicine, Kyoto, Japan.; 9 Institute for the Advanced Study of Human Biology, Kyoto University, Kyoto, Japan.; 10 Department of Genetics, Osaka University Graduate School of Medicine, Osaka, Japan.; 11 Laboratory of Intracellular Membrane Dynamics, Graduate School of Frontier Biosciences,; 12 Institute for Advanced Co-Creation Studies, and; 13 Integrated Frontier Research for Medical Science Division, Institute for Open and Transdisciplinary Research Initiatives (OTRI), Osaka University, Suita, Osaka, Japan.; 14 Telethon Institute of Genetics and Medicine (TIGEM), Pozzuoli, Naples, Italy.; 15 Medical Genetics Unit, Department of Medical and Translational Science, Federico II University, Naples, Italy.; 16 Department of Molecular and Human Genetics, Baylor College of Medicine, Houston, Texas, USA.; 17 Jan and Dan Duncan Neurological Research Institute, Texas Children’s Hospital, Houston, Texas, USA.

**Keywords:** Metabolism, Nephrology, Chronic kidney disease, Lysosomes, Obesity

## Abstract

Obesity is a major risk factor for end-stage kidney disease. We previously found that lysosomal dysfunction and impaired autophagic flux contribute to lipotoxicity in obesity-related kidney disease, in both humans and experimental animal models. However, the regulatory factors involved in countering renal lipotoxicity are largely unknown. Here, we found that palmitic acid strongly promoted dephosphorylation and nuclear translocation of transcription factor EB (TFEB) by inhibiting the mechanistic target of rapamycin kinase complex 1 pathway in a Rag GTPase–dependent manner, though these effects gradually diminished after extended treatment. We then investigated the role of TFEB in the pathogenesis of obesity-related kidney disease. Proximal tubular epithelial cell–specific (PTEC-specific) *Tfeb*-deficient mice fed a high-fat diet (HFD) exhibited greater phospholipid accumulation in enlarged lysosomes, which manifested as multilamellar bodies (MLBs). Activated TFEB mediated lysosomal exocytosis of phospholipids, which helped reduce MLB accumulation in PTECs. Furthermore, HFD-fed, PTEC-specific *Tfeb*-deficient mice showed autophagic stagnation and exacerbated injury upon renal ischemia/reperfusion. Finally, higher body mass index was associated with increased vacuolation and decreased nuclear TFEB in the proximal tubules of patients with chronic kidney disease. These results indicate a critical role of TFEB-mediated lysosomal exocytosis in counteracting renal lipotoxicity.

## Graphical abstract

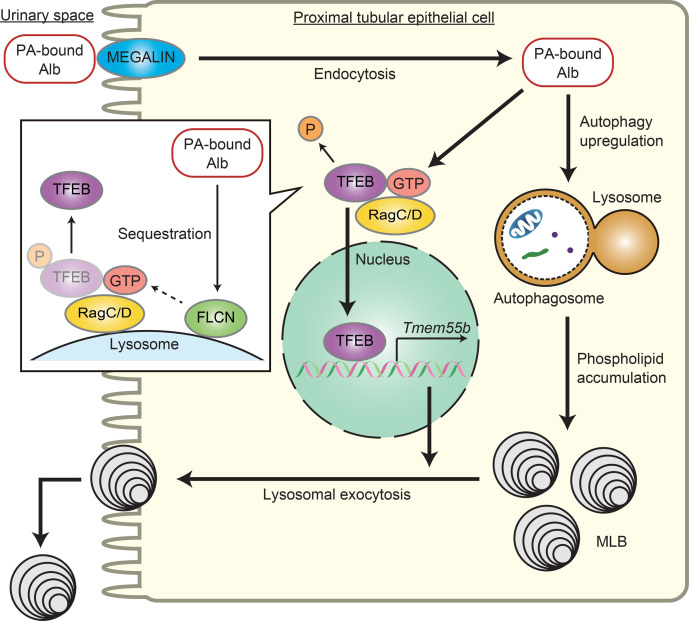

## Introduction

Obesity is an independent risk factor for kidney function decline and mortality, and the number of obese individuals has dramatically increased worldwide over the past 4 decades (1, 2). Obesity-related kidney disease has been characterized by glomerular hypertrophy and segmental sclerosis, generally referred to as “obesity-related glomerulopathy” (3). Circulating albumin–bound free fatty acids are filtered by glomeruli and endocytosed via receptors such as low-density lipoprotein receptor–related protein (LRP2)/MEGALIN in the proximal tubules (4, 5), suggesting that proximal tubular cells (PTECs) may be highly susceptible to lipid overload. In addition, obesity is characterized by adipocyte hypertrophy, resulting in secretion of pro-inflammatory saturated fatty acids, including palmitic acid (PA) (6). Although obesity-related tubular lesions have largely been overlooked relative to glomerular lesions, accumulating evidence suggests that lipid overload–induced tubular lesions, such as multilamellar bodies (MLBs) in the lysosomes, contribute to kidney dysfunction, inflammation, and fibrosis (5, 7–10). However, the regulatory mechanisms through which PTECs escape from obesity-related tubular lesions are still largely unknown. Furthermore, whether findings in murine models are applicable to obesity-related nephropathy in humans remains to be established.

Macroautophagy (hereafter referred to as autophagy) is an essential intracellular degradation system in which cytoplasmic contents are degraded in lysosomes (11). Recently, essential roles of autophagy in maintaining the function of PTECs have been elucidated in both physiological and pathological settings, such as acute kidney injury (12–14), starvation, aging, and metabolic syndrome–related diseases (15–18). Recently, we identified a novel mechanism underlying obesity-related tubular lesions: (a) vacuoles in PTECs consist of phospholipid accumulation in enlarged lysosomes; (b) lipid overload stimulates autophagy to renovate damaged plasma and organelle membranes; and (c) this autophagic activation places a burden on the lysosomal system, leading to autophagic stagnation, which manifests as phospholipid accumulation in lysosomes (10, 19).

Renal lipid accumulation is accompanied by alterations in gene expressions related to lipid metabolism (20). However, the transcriptional and regulatory control mechanisms governing obesity-related tubular lesions have not been well characterized. In the present study, we used RNA sequencing (RNA-Seq) transcriptomic analysis to identify transcription factor EB (TFEB) as a key transcriptional regulator involved in PA-induced lipotoxicity. We also investigated the role of TFEB in obesity-related tubular lesions using a model of high-fat diet–fed (HFD–fed), PTEC-specific *Tfeb*-deficient mice. Finally, we evaluated vacuole formation and TFEB activity in the tubules of nonobese and obese patients with chronic kidney disease (CKD).

## Results

### PA activates TFEB in PTECs.

**Figure 1:**
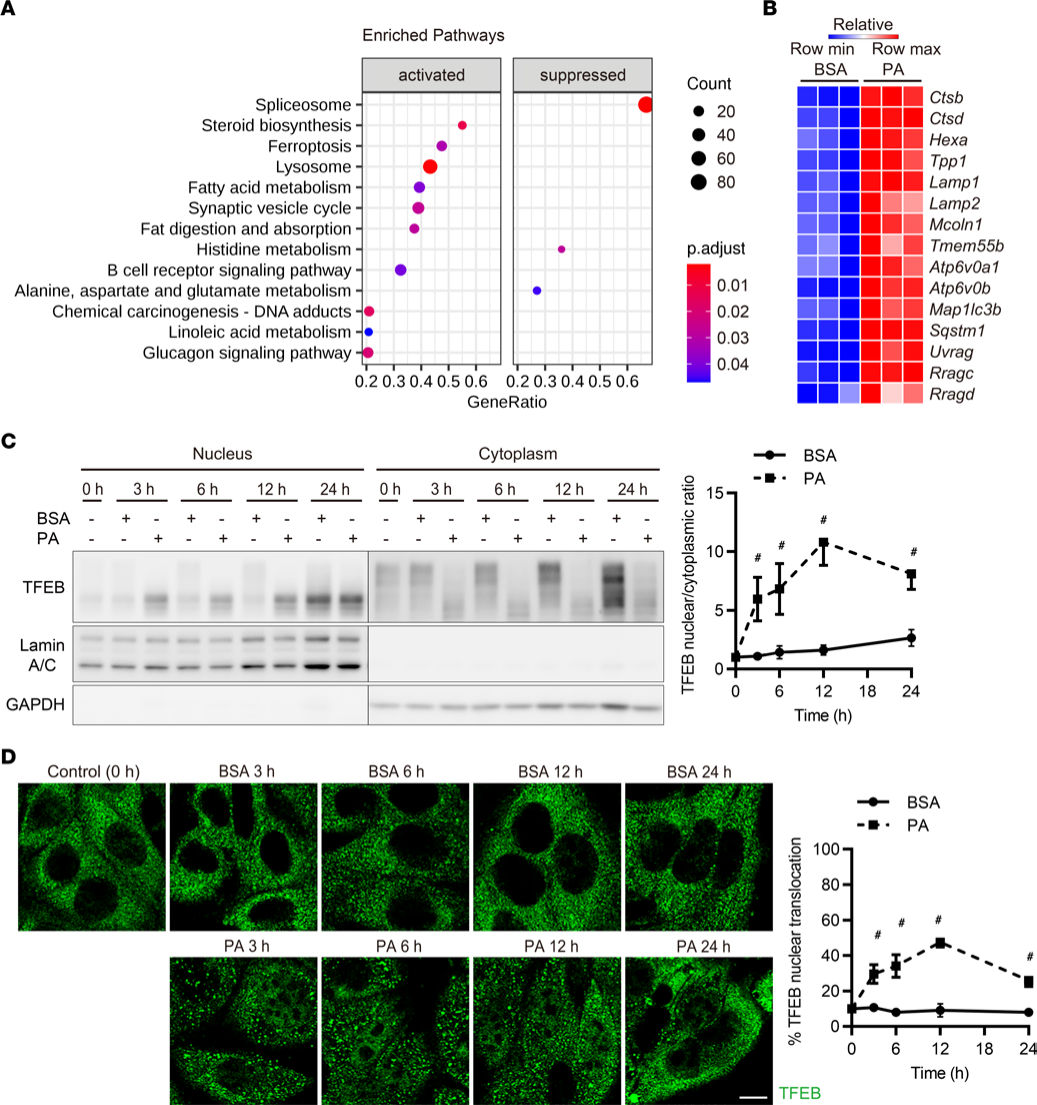
PA activates TFEB in PTECs. (**A** and **B**) We performed RNA-Seq transcriptomic analysis using cultured PTECs treated with either BSA- or PA-bound BSA for 6 hours to identify any significantly enriched pathways (*n* = 3). (**A**) Results of GSEA. Dot sizes represent the numbers of genes, while dot colors correspond to the adjusted *P* value. (**B**) A heatmap showing the relative expression of target genes. (**C**) Representative Western blot images of TFEB in nuclear and cytoplasmic fractions of cultured PTECs subjected to BSA or PA treatment for the indicated periods (*n* = 3). TFEB nuclear/cytoplasmic ratios at the indicated time points are quantified. TFEB nuclear and cytoplasmic levels were normalized for Lamin A/C and GAPDH levels, respectively. The values are normalized by the value at time 0. (**D**) Representative immunofluorescence images of TFEB in cultured PTECs subjected to BSA or PA treatment for the indicated periods (*n* = 3). The percentages of PTECs exhibiting TFEB nuclear translocation at the indicated time points are presented. Bars: 10 μm (**D**). Data are provided as means ± SEM. Statistically significant differences: ^#^*P* < 0.05 versus BSA-treated PTECs (**C** and **D**, 2-tailed Student’s *t* test).

First, to identify the regulatory pathways in PTECs during lipid overload, cultured PTECs were treated with either bovine serum albumin (BSA) or PA-bound BSA and subjected to RNA-Seq transcriptomic analysis. To determine which pathways were significantly enriched, we conducted gene set enrichment analysis (GSEA) and determined that the Kyoto Encyclopedia of Genes and Genomes (KEGG) pathway “lysosome” was significantly upregulated by PA treatment (Figure 1A and Supplemental Figure 1A; supplemental material available online with this article; https://doi.org/10.1172/jci.insight.162498DS1).

We then focused on transcription factors (TFs) that regulate lysosomal biogenesis and function during lipid overload in PTECs. To identify TFs with enriched binding within 1 kb from the transcription start sites (TSSs) of genes that are differentially expressed following PA treatment, we conducted ChIP-Atlas enrichment analysis using RNA-Seq data and compared the results with published ChIP-Atlas data sets (http://chip-atlas.org/) (21). We found that TFEB was among the top 10 of the examined TFs in terms of binding strength (Supplemental Table 1). TFEB regulates the expression of target genes bearing the coordinated lysosomal expression and regulation (CLEAR) motif, thereby regulating lysosomal biogenesis and function (22, 23). The expressions of TFEB target genes were consistently increased in PA-treated PTECs compared with those treated with BSA (Figure 1B), suggesting that PA activates TFEB in PTECs during lipid overload.

We next assessed TFEB nuclear translocation in cultured PTECs treated with BSA or PA-bound BSA. The protein levels of TFEB in the nuclear fraction were increased by PA treatment, with a peak at 12 hours, and gradually declined over extended treatment (Figure 1C). Immunostaining for TFEB verified the time-dependent changes in TFEB nuclear translocation by PA (Figure 1D). Taken together, PA activates TFEB in PTECs.

### PA activates TFEB via a Rag GTPase–dependent mechanism.

**Figure 2:**
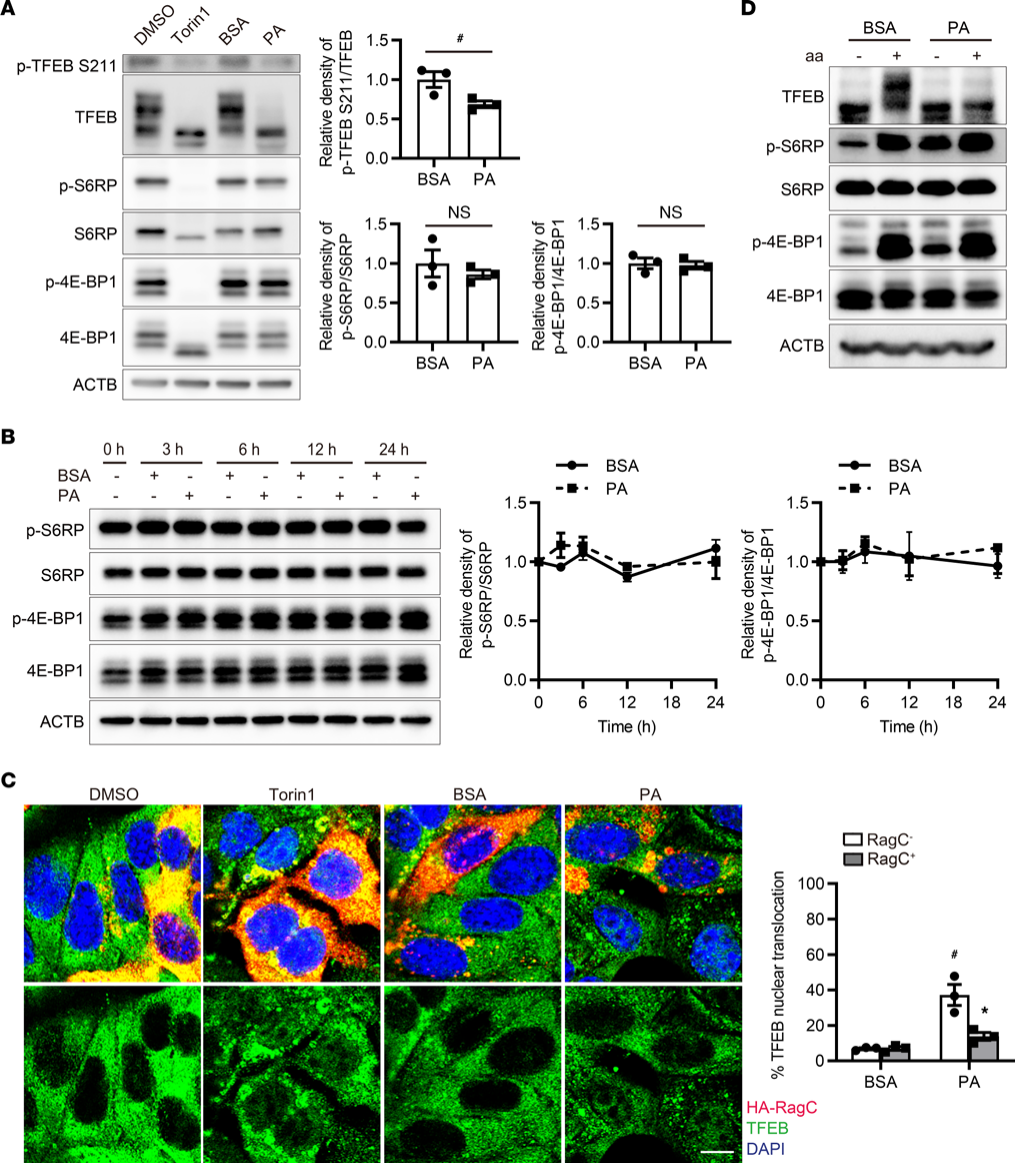
PA activates TFEB via a Rag GTPase–dependent mechanism. (**A**) Representative Western blot images of phosphorylated (p-) TFEB S211, total TFEB, p-S6RP (Ser235/236), S6RP, p–4E-BP1 (Thr37/46), and 4E-BP1 in cultured PTECs after Torin 1, BSA, or PA treatment for 6 hours (*n* = 3). (**B**) Representative Western blot images of p-S6RP (Ser235/236), S6RP, p-4E-BP1 (Thr37/46), and 4E-BP1 in cultured PTECs subjected to BSA or PA treatment for the indicated periods (*n* = 3). The values are normalized by the value at time 0. (**C**) Representative immunofluorescence images of TFEB (green) in cultured PTECs transfected with a constitutively active form of HA-tagged RagC for 48 hours, including treatment with Torin 1, BSA, or PA for the last 6 hours (*n* = 3). Cells were immunostained for HA (red) and counterstained with DAPI (blue). The percentage of PTECs exhibiting TFEB nuclear translocation was determined in wild-type PTECs (RagC^−^) and PTECs transfected with HA-tagged RagC (RagC^+^). (**D**) Representative Western blot images of TFEB, p-S6RP (Ser235/236), S6RP, p-4E-BP1 (Thr37/46), and 4E-BP1 in cultured PTECs either starved of amino acids for 60 minutes or starved for 60 minutes and then restimulated with amino acids for 30 minutes after BSA or PA treatment for 6 hours (*n* = 3). Bars: 10 μm (**C**). Data are provided as means ± SEM. Statistically significant differences: *P < 0.05 versus RagC^−^ PTECs with the same treatment; ^#^*P* < 0.05 versus BSA-treated PTECs (**A** and **B**, 2-tailed Student’s *t* test; **C**, 1-way ANOVA followed by the Tukey-Kramer test). S6RP, S6 ribosomal protein.

We explored the mechanism of PA-induced TFEB nuclear translocation and activation by focusing on mechanistic target of rapamycin kinase complex 1 (MTORC1), a key signaling pathway that negatively regulates TFEB (24). We first assessed whether PA-induced TFEB nuclear translocation is mediated by the suppression of MTORC1 activity. As seen following treatment with Torin 1, an mTOR inhibitor (25), PA dephosphorylated TFEB (Ser211) (Figure 2A). Unexpectedly, PA treatment did not affect the classical downstream signaling activities of MTORC1, such as phosphorylation of S6RP (Ser235/236) and 4E-BP1 (Thr37/46) (Figure 2, A and B). We hypothesized that RagC and RagD GTPases are involved in PA-induced TFEB dephosphorylation, since a recent study showed that TFEB is phosphorylated by MTORC1 via a substrate-specific mechanism mediated by Rag GTPases, but not by Ras homolog enriched in brain (RHEB) (26). Indeed, PA-induced TFEB nuclear translocation was inhibited by the overexpression of a constitutively active form of RagC (pRK5-HA GST RagC 75 L), while Torin 1 translocated TFEB into the nucleus even in the presence of active RagC (Figure 2C). To further explore the possible mechanism of PA-induced TFEB nuclear translocation, we focused on folliculin (FLCN), which is an amino acid–dependent GTPase-activating protein (GAP) for RagC/D (27). As expected, amino acid stimulation after starvation increased TFEB phosphorylation in BSA-treated PTECs but not in PA-treated PTECs, whereas S6RP and 4E-BP1 were similarly phosphorylated by amino acid stimulation in both BSA- and PA-treated PTECs (Figure 2D). This indicated that dysregulation of the FLCN–RagC/D axis may be involved in PA-induced TFEB activation. In addition, based on the previous reports that RagC/D activity affects MTORC1 lysosomal recruitment though to a lesser extent than RagA/B and that TFEB is phosphorylated by lysosomal MTORC1 (28, 29), we assessed whether PA affected MTOR localization. PA reduced lysosomal accumulation of MTOR (Supplemental Figure 2A), suggesting that PA may dephosphorylate TFEB partially by MTORC1 dissociation induced by inactive RagC/D.

Under certain conditions, including lysosomal perturbation, TFEB activation requires autophagy-independent autophagy-related 8 (ATG8) conjugation to the lysosomal membrane (30, 31). GABA_A_ receptor–associated protein (GABARAP; ATG8 homolog) was shown to directly bind to the FLCN/folliculin interacting protein (FNIP) complex and to sequester FLCN/FNIP to GABARAP-conjugated lysosomal membranes, which disrupts the GAP function of this complex and impairs substrate-specific, MTORC1-dependent phosphorylation of TFEB (32). Similarly, PA treatment induced the lipidation of GABARAP/microtubule-associated protein 1 light chain 3 (MAP1LC3) and the sequestration of FLCN to lysosomes (Supplemental Figure 2, B and C). PA-induced FLCN sequestration was also reduced in mouse embryonic fibroblasts (MEFs) harboring triple knockout of *Gabarap*, *Gabarapl1*, and *Gabarapl2* (GABARAP TKO) (Supplemental Figure 2D), indicating that this sequestration is dependent on GABARAP. In addition, PA-induced TFEB dephosphorylation, which was demonstrated by a downshift of the TFEB band in Western blotting due to decreased TFEB molecular weight, was impaired in MEFs harboring a deletion of *Atg7*, a component of the ATG conjugation systems essential for GABARAP/MAP1LC3 lipidation, but not in MEFs with a deletion of *Rb1cc1/Fip200*, which is required for autophagosome biogenesis (Supplemental Figure 2E). These results indicate that PA-induced TFEB dephosphorylation probably depends on lipidation of GABARAP/MAP1LC3. Using MEFs harboring double knockout of *Map1lc3a* and *Map1lc3b* (LC3 DKO) or GABARAP TKO, we investigated whether GABARAP/MAP1LC3 are required for TFEB dephosphorylation by PA. TFEB dephosphorylation was reduced in both mutant cell types after PA treatment, though the reduction was less than in MEFs harboring a deletion of *Atg7*, suggesting that both GABARAP and MAP1LC3 contribute to TFEB nuclear translocation (Supplemental Figure 2F). Together, these results suggest that PA-induced TFEB dephosphorylation and subsequent nuclear translocation are mediated by MTORC1 inhibition via a Rag-dependent mechanism.

### TFEB alleviates lipid overload–induced phospholipid accumulation in enlarged lysosomes in PTECs.

**Figure 3:**
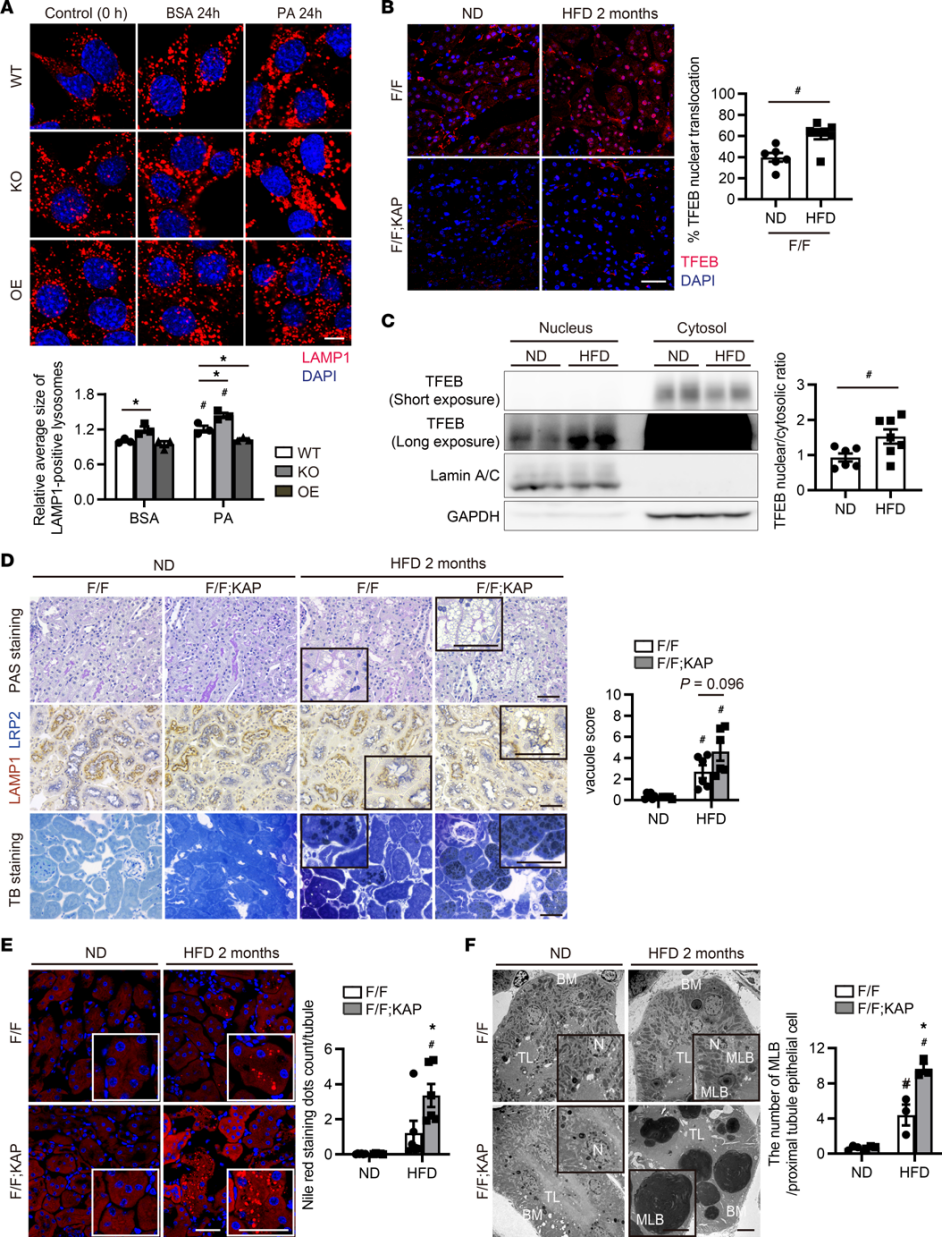
TFEB alleviates phospholipid accumulation in enlarged lysosomes during lipid overload in PTECs. (**A**) Immunofluorescence images of LAMP1 after BSA or PA treatment for 24 hours (*n* = 3). (**B**–**F**) To investigate the role of TFEB in the kidneys under lipid overload, 8-week-old *Tfeb^fl/fl^* KAP and *Tfeb^fl/fl^* control mice were fed an ND or HFD for 2 months. (**B**) Immunostaining images showing TFEB in the kidney cortical regions (*n* = 6–7). The percentage of PTECs exhibiting TFEB nuclear translocation was determined. (**C**) Western blot images of TFEB in nuclear and cytosolic fractions of kidneys (*n* = 6–7). TFEB nuclear/cytosolic ratios are shown. Nuclear and cytosolic TFEB levels were normalized for Lamin A/C and GAPDH levels, respectively. (**D** and **E**) Images of PAS staining, LAMP1 immunostaining, toluidine blue staining (**D**), and Nile red staining (**E**) in the kidney cortical regions (*n* = 5–6). Sections were immunostained for LRP2, a marker of proximal tubules (blue) (**D**) and counterstained with hematoxylin (bluish purple) (**D**) and DAPI (**E**). (**D**) Vacuole scores are shown. (**E**) The number (per proximal tubule) of Nile red–positive dots was counted. (**E**) Images of electron micrographs of the kidneys. The number of MLBs was counted (*n* = 3). Bars: 10 μm (**A**), 40 μm (**B** and **E**), 50 μm (**D**), and 5 μm (**F**). Values represent means ± SEM. Statistically significant differences: *P < 0.05 versus treatment-matched *Tfeb^fl/fl^* control littermates or wild-type PTECs; ^#^*P* < 0.05 versus nonobese mice or BSA-treated PTECs (**A**, 1-way ANOVA followed by Dunnett’s test; **D**–**F**, 1-way ANOVA followed by Tukey-Kramer test; **B** and **C**, 2-tailed Student’s *t* test). All images are representative of multiple experiments. WT, wild-type PTECs; KO, *Tfeb*-deficient PTECs; OE, *Tfeb*-overexpressing PTECs; BM, basement membrane; MLB, multilamellar body; TL, tubular lumen; N, nucleus; F/F, *Tfeb^fl/fl^* mice; F/F;KAP, *Tfeb^fl/fl^* KAP mice; PAS, periodic acid–Schiff; TB, toluidine blue.

To determine the role of PA-induced TFEB nuclear translocation, we generated PTECs that overexpressed or were deficient in *Tfeb* (Supplemental Figure 2G). PA treatment induced the accumulation of dilated lysosomes in *Tfeb*-deficient PTECs, which was restored by *Tfeb* overexpression (Figure 3A). To investigate the role of TFEB in vivo, 8-week-old, PTEC-specific *Tfeb*-deficient *Tfeb^fl/fl^* KAP (kidney androgen-regulated protein–Cre) mice and *Tfeb^fl/fl^* control mice were fed a normal diet (ND) or an HFD for 2 months, resulting in nonobese and obese mice, respectively. An immunofluorescence study and Western blot analysis demonstrated that TFEB nuclear translocation in PTECs was promoted in obese mice more significantly than in nonobese mice (Figure 3, B and C). In agreement with in vitro results, HFD-induced TFEB nuclear translocation was impaired in obese *Atg5^fl/fl^* KAP mice, whose PTECs were deficient in *Atg5*, a component of the ATG conjugation system (Supplemental Figure 2H). In the PTECs of *Tfeb^fl/fl^* KAP mice, HFD induced formation of cytosolic vacuoles, most of which exhibited lysosomal-associated membrane protein 1 (LAMP1) on the surface and were positively stained with toluidine blue (Figure 3D). Staining with Nile red, which can detect not only lipid droplets but also phospholipids (33), was more intense in PTECs of *Tfeb^fl/fl^* KAP mice (Figure 3E). Most of the Nile red–positive lipids were colocalized with LAMP1 (Supplemental Figure 3A). Electron microscopy revealed that the number of HFD-induced MLBs, which have concentric membrane layers and an electron-dense core, was increased in obese *Tfeb^fl/fl^* KAP mice (Figure 3F). Greater phospholipid accumulation in enlarged lysosomes was verified in other types of PTEC-specific *Tfeb*-deficient mice, which excluded the off-target effect of KAP-Cre (Supplemental Figure 3B). Furthermore, trehalose, a natural disaccharide (α,α-1,1-glucoside) that was reported to enhance TFEB nuclear translocation (34), increased the TFEB nuclear translocation and reduced HFD-induced formation of cytosolic vacuoles in PTECs (Supplemental Figure 3, C and D). Resveratrol, a plant-derived polyphenolic compound that is known to be a TFEB activator (35), like trehalose, also reduced the formation of cytosolic vacuoles (Supplemental Figure 3E). These data suggest that TFEB alleviates lipid overload–induced phospholipid accumulation in enlarged lysosomes in PTECs.

### PA-induced TFEB activation promotes lysosomal exocytosis of phospholipids in PTECs.

**Figure 4:**
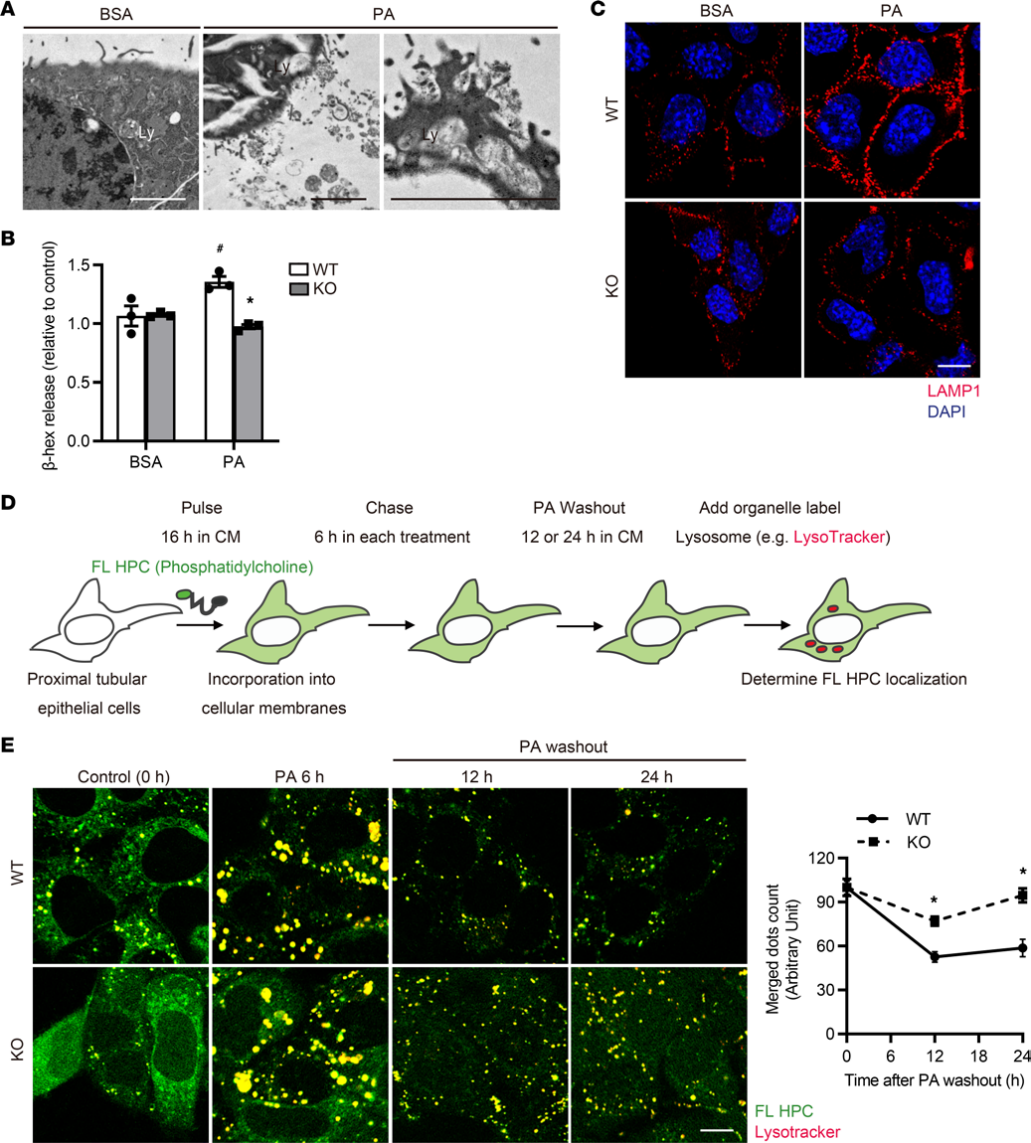
PA-induced TFEB activation promotes lysosomal exocytosis of phospholipids to prevent MLB accumulation in PTECs. (**A**–**C**) To investigate the role of TFEB on lysosomal exocytosis, wild-type and *Tfeb*-deficient PTECs were treated with BSA (0.25%, **A**, or 0.125%, **B** and **C**) or PA (0.25 mM, **A**, or 0.125 mM, **B** and **C**) for 6 hours. (**A**) Electron micrographs of wild-type PTECs (*n* = 2). (**B**) β-Hexosaminidase activity in the culture supernatant of PTECs relative to the total activity (*n* = 3). (**C**) Immunofluorescence images of nonpermeabilized PTECs show only LAMP1 that is exposed on the plasma membrane (*n* = 3). (**D** and **E**) To investigate the trafficking of phospholipids, a fluorescent fatty acid pulse-chase assay was performed. (**D**) Schematic illustration of pulse-chase assay. FL HPC–loaded wild-type and *Tfeb*-deficient PTECs were chased after treatment with either 0.125% BSA or 0.125 mM PA for 6 hours, and the subcellular localization of FL HPC was determined by staining with LysoTracker Red for 0, 12, or 24 hours after PA washout. (**E**) To measure phospholipid accumulation in lysosomes, the number of dots indicating staining for both phospholipids and lysosomes was counted (*n* = 3). Bars: 5 μm (**A**) and 10 μm (**C** and **E**). Data are provided as means ± SEM. Statistically significant differences: *P < 0.05 versus wild-type PTECs with the same treatment; ^#^*P* < 0.05 versus BSA-treated PTECs (**B**, 1-way ANOVA followed by the Tukey-Kramer test; **E**, 2-tailed Student’s *t* test). All images are representative of multiple experiments. Ly, lysosome; WT, wild-type PTECs; KO, *Tfeb*-deficient PTECs; DIC, differential interference contrast.

We next assessed HFD-fed, PTEC-specific *Tfeb*-deficient mice in terms of lysosomal biogenesis and function, since these are critically impaired in other cell types in several disease models (36–39). Unexpectedly, lysosomal biogenesis and proteolytic activity, as assessed by the protein levels of LAMP1 and mature cathepsin D (CTSD), showed no significant differences between obese *Tfeb^fl/fl^* and *Tfeb^fl/fl^* KAP mice (Supplemental Figure 4A). Similarly, there was no significant difference in lysosomal biogenesis or proteolytic activity between control and *Tfeb*-deficient PTECs after PA treatment (Supplemental Figure 4B). A recent study reported that TFEB regulates lysosomal exocytosis, and induction of lysosomal exocytosis by TFEB overexpression has been shown to rescue pathologic storage and restore normal cellular morphology in lysosomal storage disease (LSD) (40, 41). Thus, we deduced that TFEB-mediated lysosomal exocytosis is involved in phospholipid accumulation in PTEC lysosomes, since it can be regarded as an acquired form of LSD (8, 10). Electron microscopy analysis revealed that lysosomal contents were released outside the plasma membrane during PA treatment, indicating that PA induces lysosomal exocytosis (Figure 4A). PA significantly increased the release of the lysosomal enzyme *N*-acetyl-β-d-glucosaminidase (β-hexosaminidase) in the culture supernatant of wild-type PTECs, without significantly altering lactate dehydrogenase (LDH) release, an indicator of cell death, and this increase was completely abolished by TFEB deficiency (Figure 4B and Supplemental Figure 4C). Lysosomal exocytosis was verified by staining nonpermeabilized cells with anti-LAMP1 antibody and observing exposed LAMP1 on the plasma membrane (Figure 4C). To further investigate the trafficking of phospholipids, we performed a pulse-chase assay. Cultured PTECs were initially labeled with trace amounts of phosphatidylcholine that was fluorescence-tagged at its fatty acid tail (FL HPC) for 16 hours and chased with each treatment for 6 hours (Figure 4D) (10). Colocalization of FL HPC and LysoTracker Red was increased in PA-treated PTECs regardless of TFEB deficiency, indicating that PA promotes phospholipid redistribution into the lysosomes for autophagic degradation. PA significantly increased the number of FL HPC–positive and LysoTracker-negative dots outside the cell border in wild-type PTECs, but the increase was almost completely abolished by TFEB deficiency, suggesting that more phospholipids were released by PA-treated PTECs and this release was dependent on TFEB (Supplemental Figure 4D). In addition, to determine the impact of this release on the clearance of lysosomal phospholipid accumulation, we observed PA-treated PTECs for up to 24 hours after PA washout. Dots indicating colocalized phospholipids and lysosomes were decreased in wild-type PTECs, but the decrease was completely abolished by TFEB deficiency (Figure 4E). Collectively, PA-induced TFEB activation promotes lysosomal exocytosis of phospholipids in PTECs.

### MLBs are excreted into the tubular lumen in obese mice.

**Figure 5:**
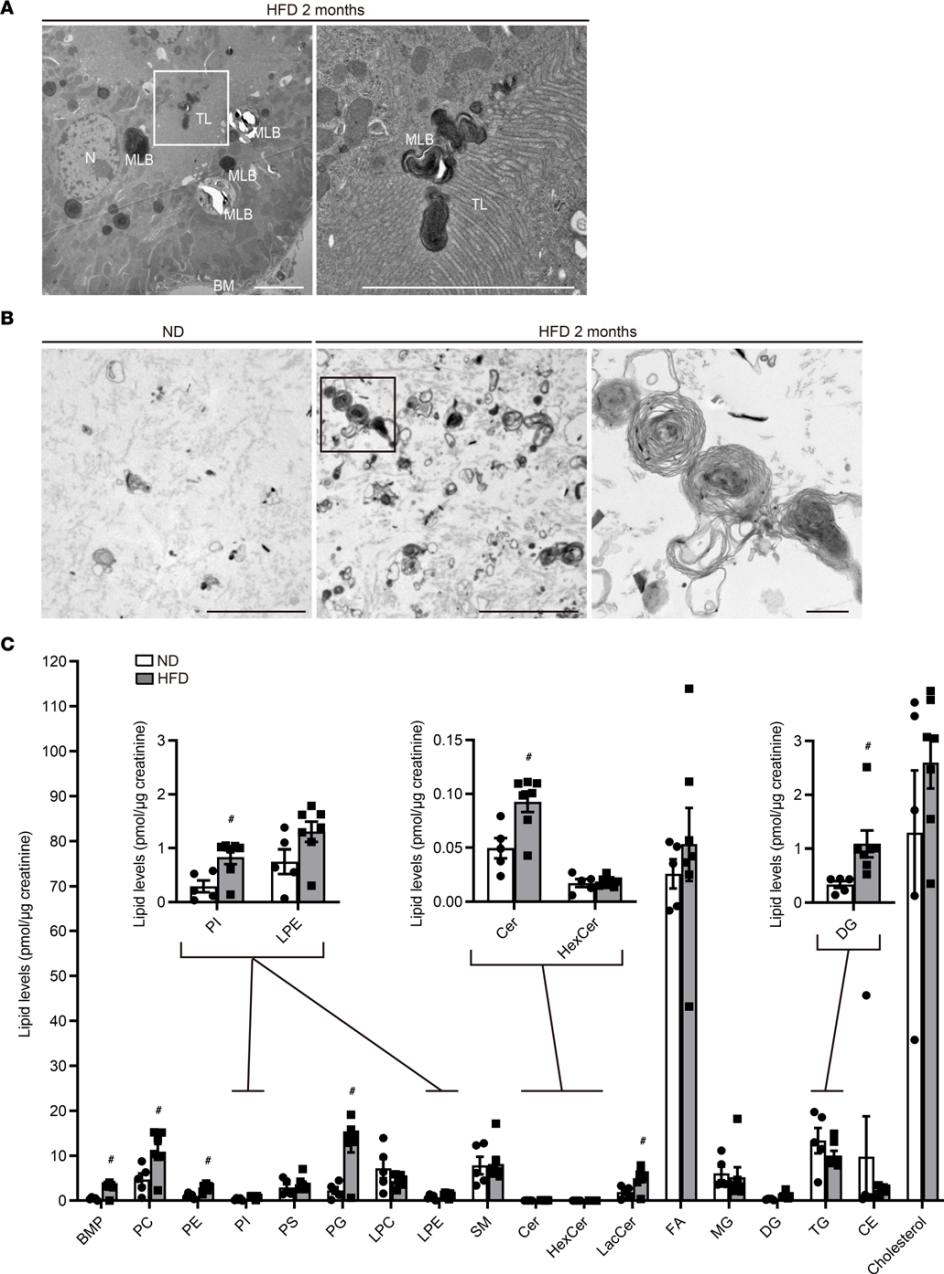
MLBs are released from the apical membrane of PTECs into the tubular lumen in obese mice. (**A** and **B**) Representative electron micrographs of the kidneys (**A**) and the urine pellets (**B**) of nonobese and obese *Tfeb^fl/fl^* mice (*n* = 2–3). (**C**) Urinary lipidomics of nonobese and obese mice (*n* = 5 or 7). Data are presented as the total amount of each lipid species normalized to urine creatinine concentration. Bars: 5 μm (**A** and **B**) and 500 nm (**B**, magnified image). Values represent means ± SEM. Statistically significant differences: ^#^*P* < 0.05 versus nonobese mice (**C**, 2-tailed Student’s *t* test). BM, basement membrane; MLB, multilamellar body; TL, tubular lumen; N, nucleus; BMP, bis(monoacylglycerol) phosphate; PC, phosphatidylcholine; PE, phosphatidylethanolamine; PI, phosphatidylinositol; PS, phosphatidylserine; PG, phosphatidylglycerol; LPC, lysophosphatidylcholine; LPE, lysophosphatidylethanolamine; SM, sphingomyelin; Cer, ceramide; HexCer, hexose ceramide; LacCer, lactosylceramide; FA, fatty acid; MG, monoacylglycerol; DG, diacylglycerol; TG, triacylglycerol; CE, cholesterylester.

Next, we examined whether lysosomal exocytosis of MLBs actually occurs in obese mice. Electron microscopy revealed that MLBs were released from the apical membrane into the tubular lumen in PTECs (Figure 5A). MLBs were consistently detected in the urine of obese mice but rarely in that of nonobese mice (Figure 5B). The size of released MLBs was much larger than 150 nm, the maximum size of exosomes that originate from multivesicular bodies (42), suggesting that the MLBs in the tubular lumen had mainly originated from enlarged lysosomes rather than from intracellular multivesicular bodies. In Fabry disease, an LSD, multilamellar inclusions within lysosomes are found predominantly in podocytes and distal tubules (43). Therefore, we investigated whether cells other than PTECs contained MLBs. MLBs were not observed in distal tubules or podocytes (Supplemental Figure 5, A and B), suggesting that MLBs were derived from PTECs. Moreover, lipidomic analysis demonstrated that the urinary levels of many phospholipids, such as bis(monoacylglycerol) phosphate (BMP), a lysosomal phospholipid whose tissue levels are increased in patients with phospholipidosis (44), were significantly increased in obese mice compared with nonobese mice (Figure 5C and Supplemental Figure 5, C–F). These data suggest that MLBs are excreted into the tubular lumen in obese mice.

### TFEB promotes apical transport of lysosomes in PTECs of obese mice.

**Figure 6:**
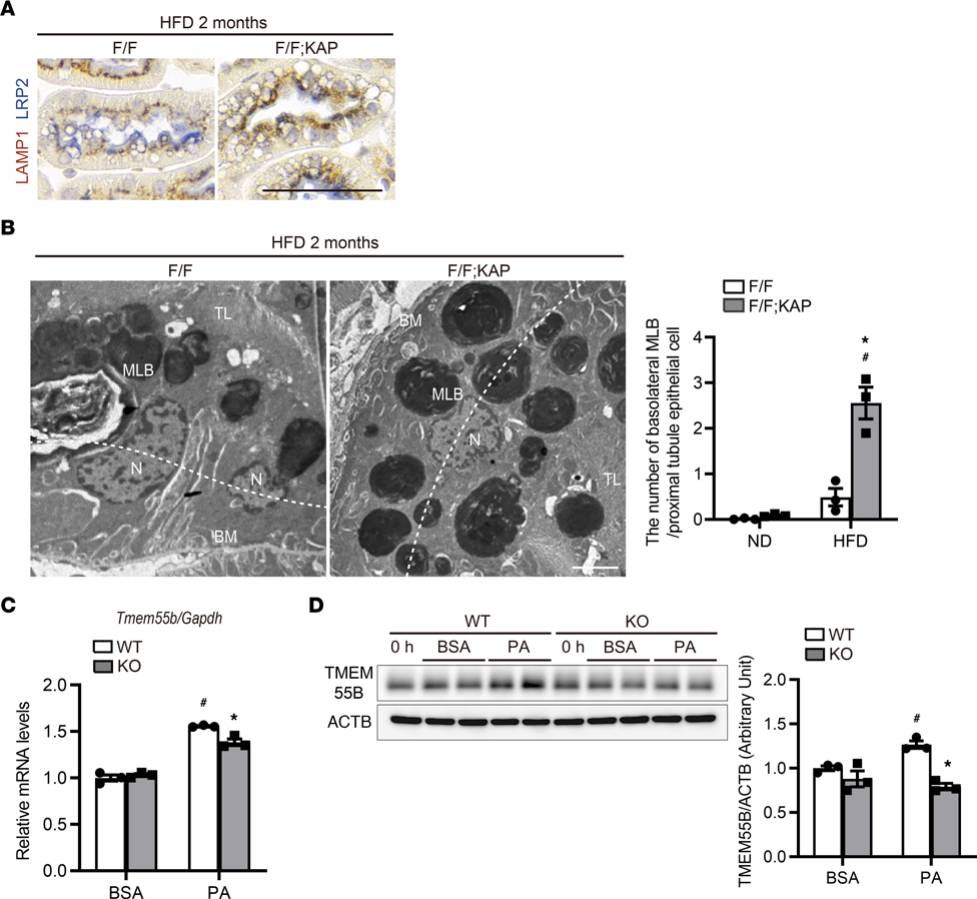
TFEB promotes apical transport of lysosomes in PTECs of obese mice. (**A** and **B**) Representative images showing immunostaining for LAMP1 (**A**) and electron microscopy (**B**) of the kidney cortical regions of obese *Tfeb^fl/fl^* and *Tfeb^fl/fl^* KAP mice. (**A**) Sections were immunostained for LRP2, a marker of proximal tubules (blue) and counterstained with hematoxylin (bluish purple) (*n* = 5–6). (**B**) Basolateral MLBs were defined if they were located outside the white dotted line connecting the nuclei. The number of basolateral MLBs under each condition was counted in at least 20 PTECs (*n* = 3). (**C** and **D**) mRNA (**C**) and protein (**D**) levels of *Tmem55b* relative to *Gapdh* (**C**) and ACTB (**D**), respectively, in wild-type and *Tfeb*-deficient PTECs 6 hours after BSA or PA treatment (*n* = 3). The values are normalized by the mean value of BSA-treated, wild-type PTECs. Bars: 50 μm (**A**) and 5 μm (**B**). Values represent means ± SEM. Statistically significant differences: *P < 0.05 versus treatment-matched *Tfeb^fl/fl^* control littermates or wild-type PTECs; ^#^*P* < 0.05 versus nonobese mice or BSA-treated PTECs (**B**–**D**, 1-way ANOVA followed by the Tukey-Kramer test). BM, basement membrane; MLB, multilamellar body; TL, tubular lumen; N, nucleus. F/F, *Tfeb^fl/fl^* mice; F/F;KAP, *Tfeb^fl/fl^* KAP mice; WT, wild-type PTECs; KO, *Tfeb*-deficient PTECs.

Under physiological conditions, LAMP1-positive lysosomes are localized underneath the LRP2-positive apical plasma membrane in PTECs (Figure 3D and Supplemental Figure 3B), and this was observed in obese *Tfeb^fl/fl^* mice. By contrast, LAMP1-positive vacuoles had a scattered distribution in obese *Tfeb^fl/fl^* KAP mice (Figure 6A). Ultrastructurally, basolateral localization of MLBs was also observed in obese *Tfeb^fl/fl^* KAP mice (Figure 6B). Based on these observations, we postulated that TFEB regulates dynein-dependent transport of lysosomes in obese mice. Lysosomes move along microtubules, whose geometries vary according to cell type. In unpolarized cells, microtubule minus ends are located around the nucleus while plus ends are situated around the cell periphery. However, polarized epithelial cells such as PTECs contain a polarized microtubule network that runs mostly in the apicobasal direction: microtubule minus ends are positioned on the apical side and the plus ends are found on the basolateral side (45). We measured the mRNA and protein levels of *Tmem55b/Pip4p1*, a TFEB downstream gene involved in retrograde transport toward microtubule minus ends (46). Upregulation of transmembrane protein 55b (TMEM55B) was observed after treatment with PA in wild-type PTECs, but the increase was less pronounced in *Tfeb*-deficient PTECs (Figure 6, C and D). These data suggest that TFEB promotes apical transport of lysosomes in PTECs under lipid overload.

### TFEB deficiency stagnates autophagic flux and increases vulnerability to ischemia/reperfusion kidney injury during HFD treatment.

**Figure 7:**
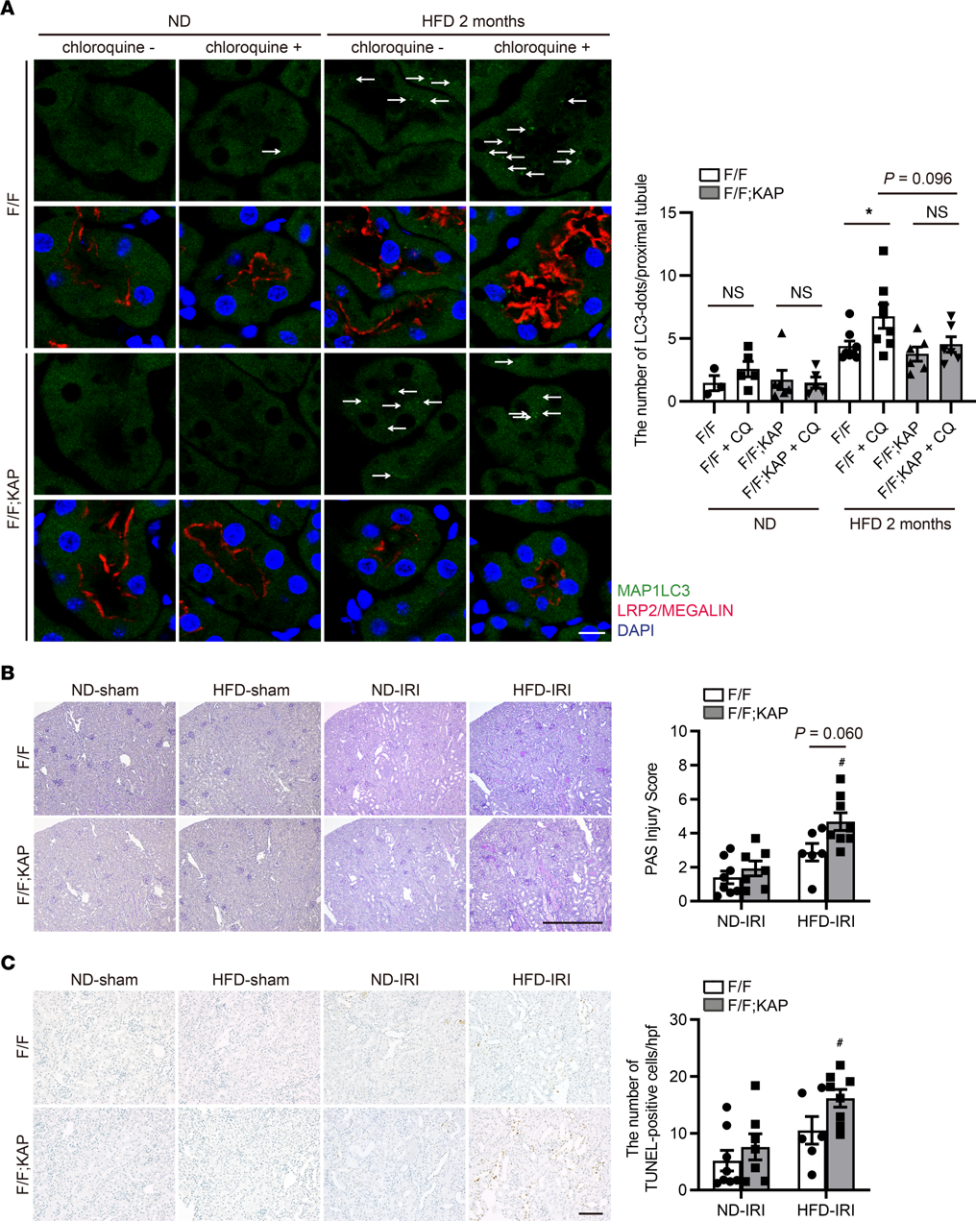
TFEB deficiency stagnates autophagic flux and enhances vulnerability to ischemia/reperfusion injury during HFD treatment. (**A**) Autophagic flux was assessed by counting the number of GFP-positive dots in the proximal tubules of nonobese or obese GFP-MAP1LC3 transgenic *Tfeb^fl/fl^* or *Tfeb^fl/fl^* KAP mice with or without chloroquine administration (*n* = 3–6 in the nonobese group and 6–8 in the obese group). The number of GFP-positive dots per proximal tubule under each condition was counted in at least 10 high-power fields (original magnification, ×600) (each high-power field contained 10–15 proximal tubules). Representative images are presented. (**B** and **C**) Representative images of PAS (**B**) and TUNEL (**C**) staining of the kidney cortical regions of nonobese and obese *Tfeb^fl/fl^* or *Tfeb^fl/fl^* KAP mice 2 days after unilateral IR or sham operation (*n* = 6–8 in each group). Sections were immunostained for LRP2, a marker of proximal tubules (blue) (**A**) and counterstained with DAPI (**A**) and methyl green (blue/green) (**C**). (**B**) The tubular injury score is shown. (**C**) The number of TUNEL-positive PTECs was calculated in at least 10 high-power fields. Bars: 10 μm (**A**), 500 μm (**B**), and 100 μm (**C**). Data are provided as means ± SEM. Statistically significant differences: *P < 0.05 versus mice with no chloroquine treatment; ^#^*P* < 0.05 versus nonobese mice (**A**, 2-tailed Student’s *t* test; **B** and **C**, 1-way ANOVA followed by the Tukey-Kramer test). Arrows indicate GFP-MAP1LC3 dots. CQ, chloroquine; F/F, *Tfeb^fl/fl^* mice; F/F;KAP, *Tfeb^fl/fl^* KAP mice.

Phospholipid accumulation in lysosomes stagnates autophagy (19). To assess autophagic flux in vivo, GFP-MAP1LC3 transgenic *Tfeb^fl/fl^* and *Tfeb^fl/fl^* KAP mice fed with an ND or HFD were administered chloroquine 6 hours before euthanasia (16). GFP-positive dots that represent autophagosomes were rarely observed in chloroquine-free nonobese *Tfeb^fl/fl^* or *Tfeb^fl/fl^* KAP mice, and the numbers of these dots remained unchanged by chloroquine administration, suggesting that autophagic activities in both nonobese mice were low. By contrast, a substantial number of dots were observed in PTECs of chloroquine-free obese *Tfeb^fl/fl^* and *Tfeb^fl/fl^* KAP mice. Chloroquine administration significantly increased the number of dots in obese *Tfeb^fl/fl^* mice, whereas this number remained unchanged in obese *Tfeb^fl/fl^* KAP mice, suggesting that autophagy flux was high in obese *Tfeb^fl/fl^* mice but was stagnated by TFEB deficiency (Figure 7A). TFEB deficiency also reduced the formation of autophagosomes, as indicated by the relatively fewer dots in obese *Tfeb^fl/fl^* KAP mice even after chloroquine administration. Electron microscopy validated the results of this immunofluorescence study (Supplemental Figure 6A).

Next, since our previous study showed that autophagy in PTECs counteracts ischemia/reperfusion (IR) injury (10), we assessed whether the vulnerability toward IR injury was affected by TFEB deficiency in nonobese and obese mice. No significant difference in kidney injury was observed between nonobese *Tfeb^fl/fl^* and *Tfeb^fl/fl^* KAP mice following IR, but we observed more severe injury with massive tubular sediments and vacuolation in obese *Tfeb^fl/fl^* KAP mice compared with obese *Tfeb^fl/fl^* mice (Figure 7B). Moreover, the number of TUNEL-positive tubular cells was prominently increased in obese *Tfeb^fl/fl^* KAP mice (Figure 7C). Both impaired autophagic flux and reduced cell viability following PA treatment were consistently exacerbated in *Tfeb*-deficient PTECs and rescued by TFEB overexpression (Supplemental Figure 6, B and C). Taken together, TFEB deficiency stagnates autophagic flux and enhances vulnerability toward IR injury during HFD treatment.

### Toxic lipids released from MLBs cause tubular injury after IR.

We examined whether MLBs themselves cause lipotoxicity. MLB formation can be regarded as a process for sequestering potentially harmful metabolites, such as fatty acyl-CoA, free cholesterol, diacylglycerol, and ceramides, which suppresses toxicity (47). Indeed, HFD-fed, PTEC-specific *Tfeb*-deficient mice exhibited no obvious worsening of inflammation or fibrosis despite marked MLB accumulation (Supplemental Figure 6D). Then, we speculated that giant MLBs would be more susceptible to lysosomal membrane permeabilization (LMP) under acute injury, resulting in lipotoxicity because of the release of lipids from the lysosomal lumen to the cytosol. Indeed, electron microscopy revealed that lysosomal membranes of giant MLBs were interrupted in obese *Tfeb^fl/fl^* KAP mice (Supplemental Figure 7A). Moreover, GALECTIN-3 dots, a sensitive marker of LMP (48), exhibited significant accumulation around enlarged vacuoles corresponding to the giant MLBs (Supplemental Figure 7B).

Next, we examined the lysosomal lipid composition in the kidneys of obese mice to investigate which lipids in MLBs cause lipotoxicity. Lipidomic analysis of the lysosomal fraction in kidneys revealed an increase in BMP and in toxic lipids such as diacylglycerol and ceramides (Supplemental Figure 7, C and D). This result was in good agreement with that obtained by urine lipidomics. We next focused on a lysosomal ceramide that induces apoptosis (49). When the cell-permeable analog C_2_-ceramide was administered to PTECs in vitro, cell viability was reduced (Supplemental Figure 7E). These data suggest that toxic lipids released from MLBs into the cytosol cause severe tubular toxicity under acute kidney injury in obese mice.

### Higher BMI is associated with increased vacuolation and decreased TFEB nuclear localization in PTECs of patients with CKD.

**Figure 8:**
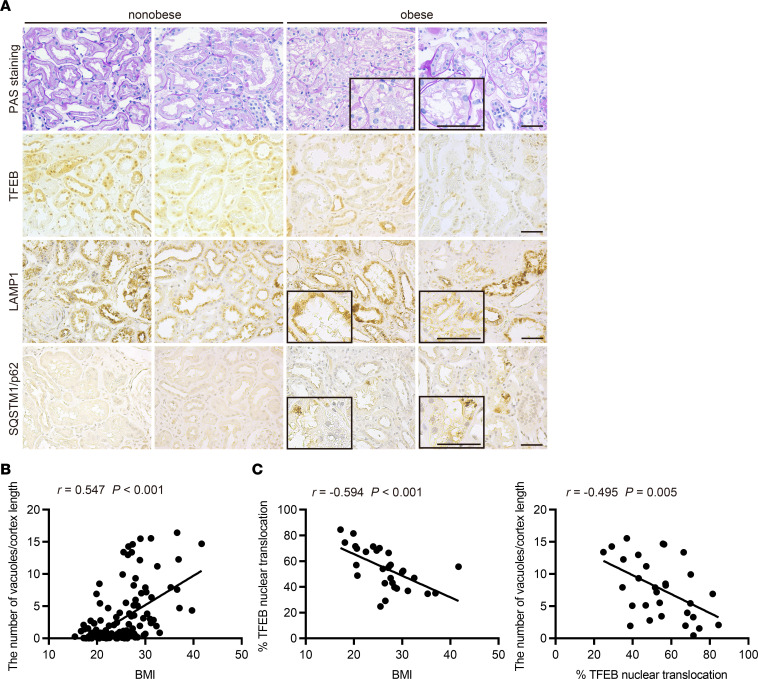
Higher BMI is associated with increased vacuolation and decreased nuclear TFEB in PTECs of patients with CKD. (**A**) Representative images of PAS staining and immunohistochemical staining for TFEB, LAMP1, and SQSTM1/p62 on kidney specimens obtained from obese and nonobese patients. Specimens were counterstained with hematoxylin. Magnified images are shown in the insets (original magnification, ×400). Bars: 50 μm. (**B** and **C**) Correlation between BMI and the severity of vacuolar formation (**B**) (*n* = 146) and between the percentage of PTECs exhibiting TFEB nuclear translocation and either BMI or the number of vacuoles (**C**) (*n* = 30). Relationships were examined using Pearson’s correlation and the corresponding *P* values. SQSTM1, sequestosome 1.

We next analyzed kidney biopsy samples from obese and nonobese patients with CKD. In contrast with obese mice, nuclear TFEB localization was suppressed in patients who were obese (Figure 8A). Moreover, many patients who were obese exhibited LAMP1-positive vacuoles and SQSTM1/p62 accumulation in PTECs, findings that were rarely observed in nonobese patients (Figure 8A). There was a significant positive correlation between the severity of vacuolar formation and BMI (Figure 8B and Supplemental Table 2) and a negative correlation between the percentage of PTECs exhibiting TFEB nuclear translocation and both the BMI and number of vacuoles (Figure 8, A and C, and Supplemental Table 3). These results suggest that decreased TFEB activity may be associated with increased vacuolation and impaired autophagy flux in PTECs in obese patients.

## Discussion

**Figure 9:**
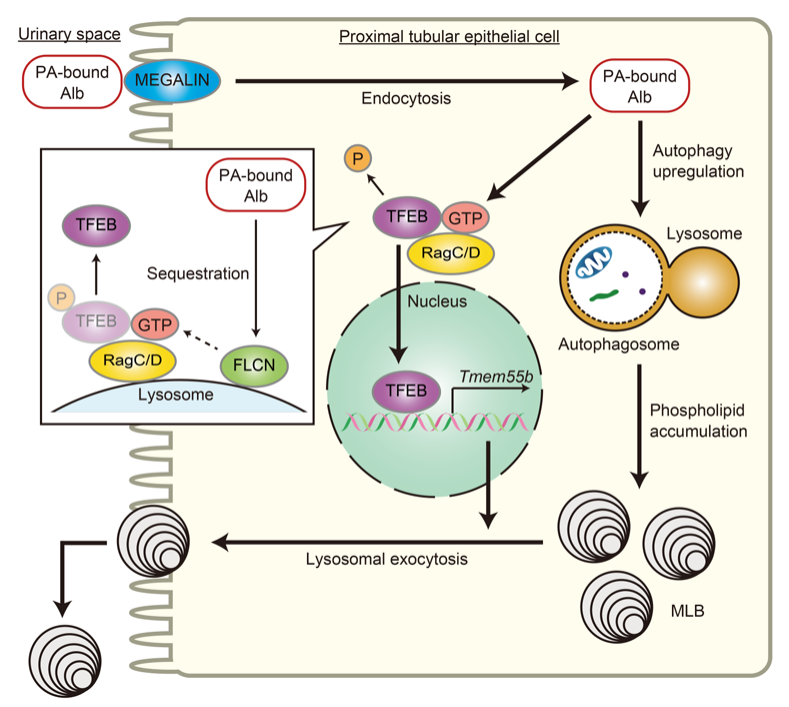
TFEB-mediated lysosomal exocytosis of MLBs is involved in ORT. Schematic illustration of this study. PTECs retrieve albumin-bound PA from the glomerular filtrate via megalin-mediated albumin endocytosis, which is delivered to lysosomes for degradation. PA strongly induces autophagy, which mobilizes phospholipids from cellular membranes to lysosomes, resulting in MLB accumulation. On the other hand, PA promotes TFEB nuclear translocation via Rag GTPase inactivation by sequestration of FLCN to the lysosomal membrane; this mediates lysosomal exocytosis of phospholipids into the apical tubular space to prevent MLB accumulation and counteract lipotoxicity.

In this study, we demonstrated 3 main findings. First, lipid overload promotes TFEB nuclear translocation via Rag-dependent inhibition of the MTORC1 pathway in PTECs, which serves to prevent MLB accumulation through trafficking of lysosomes and lysosomal exocytosis. Second, TFEB deficiency stagnates autophagic flux and enhances vulnerability to IR injury in obese mice. Third, insufficient TFEB activity and impaired autophagic flux may be involved in obesity-related vacuolar lesions in patients with CKD. These results provide fundamental insights into the way in which TFEB transcriptionally controls MLB accumulation in PTECs. MLB formation can be regarded as a process to sequester potentially toxic metabolites in a less toxic manner (47). However, mice with HFD-induced obesity and massive MLB accumulation have no capacity to augment autophagy and exhibit increased sensitivity to LMP in response to additional stressors such as ischemia. Thus, MLB accumulation is a hallmark of lysosomal stress as well as lipotoxicity, and TFEB-mediated lysosomal exocytosis of phospholipids is important to alleviate autophagic stagnation and maintain PTEC integrity against lipotoxicity. We herein propose the disease concept “obesity-related proximal tubulopathy (ORT)” and summarize it in the simple schematic drawing (Figure 9).

We and other groups have previously proposed that MLB formation in the kidney is promoted by endocytosis (5), oxysterols and impaired cholesterol trafficking (8), and upregulated autophagy (10) but hindered by enhancement of the AMP-activated protein kinase pathway (7). TFEB, which we identified as a key factor that downregulated MLB formation in the present study, may be involved in these previously proposed processes. The LRP2-mediated endocytosis of glomerulus-filtered (lipo)toxic substances is involved in MLB formation, since this formation is effectively blocked by genetic LRP2 ablation in HFD-fed obese mice (5). TFEB induces the expression of a number of core endocytic genes, including *Cav2*, *Clta*, *Eea1*, and *Rab5*, which results in increased endocytic rates under baseline and amino acid starvation conditions (50). However, our finding that genetic deletion of *Tfeb* instead exacerbates phospholipid accumulation indicates that TFEB has little effect on endocytosis during MLB formation. Autophagy is also involved in the formation of MLBs through the mobilization of phospholipids from cellular membranes to lysosomes, since ablation of autophagy prevents MLB formation (10). TFEB orchestrates the expression of a broad number of autophagy genes (51); however, our in vivo autophagy flux assay revealed that TFEB deficiency stagnates autophagic flux rather than reducing autophagosome formation (Figure 7A and Supplemental Figure 5A), suggesting that TFEB has little influence on autophagy induction.

Lysosomal biogenesis and maintenance of lysosomal degradative capacity are 2 of the most intensively studied roles of TFEB (22, 52). It is possible that enhancing lysosomal function by TFEB reduces MLB accumulation. Indeed, accumulating evidence has indicated the critical impact of TFEB-mediated lysosomal biogenesis in human diseases (36–39). Unexpectedly, however, lysosomal biogenesis and function were not altered by *Tfeb* deficiency in PTECs in our mouse model. This may be attributed to differences in cell or tissue types, observation periods, and, more likely, a wide range of roles and functions that are regulated by TFEB during the process of cellular clearance. TF binding to IGHM enhancer 3, another member of the microphthalmia/transcription factor E family, might compensate for the chronic loss of TFEB, as demonstrated in the liver (36).

We demonstrated that lipid overload is associated with TFEB involvement in lysosomal exocytosis, which serves to prevent MLB accumulation. During lysosomal exocytosis, lysosomes fuse with the plasma membrane and release their contents extracellularly; these contents then play an important role in clearing cellular debris and repairing the plasma membrane (53). The trafficking of lysosomes along microtubules is important for lysosomal exocytosis; many in vitro studies have revealed that this process requires that lysosomes migrate from the perinuclear region to the cell surface via kinesin motor proteins (antegrade transport). However, the roles and mechanisms of lysosomal exocytosis in polarized epithelia are open questions. One study demonstrated that in polarized Madin-Darby canine kidney cells, lysosomal exocytosis occurs preferentially at the basolateral membrane in response to localized calcium influx (54). In contrast, our data revealed that lipid overload induced TFEB-mediated lysosomal exocytosis of phospholipids at the apical membrane in PTECs. In our study, the lysosomal transmembrane protein TMEM55B (which has CLEAR motifs in its promoter region) may be involved in lysosomal positioning to facilitate lysosomal exocytosis, which is in contrast to recent in vitro findings showing that TFEB-dependent upregulation of TMEM55B by starvation induced dynein-dependent transport of lysosomes toward the perinuclear region (retrograde transport), contributing to the stimulation of effective autophagic flux (46). This discrepancy may be due to polarity, since polarized epithelia have a polarized microtubule network that runs mostly in the apicobasal direction: microtubule minus ends are situated near the apical side while the plus ends are located near the basolateral side (45). Alternatively, the discrepancy may be caused by the use of different autophagy inducers (starvation vs. lysosomal stress [lipid overload]). Indeed, a recent study showed that induction of lysosomal exocytosis by TFEB overexpression rescues pathologic storage and restores normal cellular morphology both in vitro and in vivo in LSDs (41). What triggers TFEB activation in response to lipid overload? We showed that TFEB activation by PA treatment was dependent on substrate-specific inactivation of MTORC1, which is consistent with a recent report (26). Due to this mechanism, the phosphorylation of TFEB, unlike that of classical MTORC1 pathway components such as S6K and 4E-BP1, is strictly dependent on the amino acid–mediated activation of RagC/D GTPases but is insensitive to RHEB activity growth factors induce. The sequestration of FLCN to lysosomes and the lipidation of GABARAP/MAP1LC3 were consistently observed after PA treatment (Supplemental Figure 2, B and C).

Contrary to TFEB activation during lipid overload in both in vitro and in vivo (2-month HFD in mice), the percentage of PTECs exhibiting TFEB nuclear translocation decreased in obese patients. Although further studies are needed to fully explain this discrepancy, one possible reason is that longer PA exposure may somehow decrease the sequestration of FLCN/FNIP to lysosomes (Supplemental Figure 2C), which prevents nuclear retention of TFEB by altering Rag C/D activity. Otherwise, many confounding factors related to obesity, such as high levels of blood glucose, insulin, amino acids, and growth factors, may influence the MTORC1 pathway (especially RagC/D GTPases). Indeed, longer term lipid overload (HFD for 10 months) blunted TFEB nuclear translocation in obese mice (Supplemental Figure 8). These findings indicate that insufficient TFEB activity with increased tubular vacuolar lesions may be a principal determinant of decline in renal function in obese patients. Thus, activating TFEB may be an attractive treatment strategy for ORT. However, kidney-specific TFEB overexpression in transgenic mice results in papillary carcinomas with severe cystic pathology via the WNT pathway (55). Therefore, targeted activation of TFEB downstream genes that are involved in lysosomal exocytosis, such as TMEM55B, would be required. Moreover, to assess the severity of ORT, less invasive methods are needed. Interestingly, we observed that urinary excretion of di-docosahexaenoyl (22:6)–BMP, a noninvasive biomarker of phospholipidosis (44), was increased in obese mice (Supplemental Figure 5), suggesting that it may be useful as a diagnostic and prognostic marker for ORT.

In conclusion, we report that TFEB-mediated lysosomal exocytosis alleviates renal lipotoxicity and that insufficient TFEB activity may play a critical role in the pathogenesis of human ORT. These data shed light on the mechanisms underlying phospholipid accumulation and provide possible therapeutic strategies for ORT.

## Methods

### Cell culture.

Immortalized wild-type PTEC lines were cultured in accordance with previously described methods (12). PTECs were transfected with pRK5-HA GST RagC 75 L plasmids (Addgene plasmid 19305) using Lipofectamine 3000 reagent (Thermo Fisher Scientific, L3000015) for 48 hours after plating. *Tfeb*-deficient PTEC lines were generated using CRISPR guide RNAs (gRNAs). Annealed gRNA oligonucleotides were inserted into vector px458, and the gRNA constructs were transfected into PTECs using ViaFect (Promega, E4981) transfection reagent. GFP-positive single cells were sorted by FACS into 96-well plates. Candidate single-clone colonies were verified by immunoblotting using specific antibodies (see *Antibodies*) and genomic DNA sequencing. The gRNA sequence was as follows: TFEB, 5′ CATGCAGCTCATGCGGGAGC 3′. *Tfeb*-overexpressing PTEC lines were generated by transfection with mNeonGreen-tagged TFEB as described previously (30). PTECs were grown in low-glucose DMEM (Nacalai Tesque, 08456-65) with 5% FBS and 1% penicillin/streptomycin (MilliporeSigma, P4333). *Atg7*^+/+^ and *Atg7*^–/–^ MEF cell lines were provided by M. Komatsu (Juntendo University, Tokyo, Japan) (56); *Fip200* WT and *Fip200*-KO MEF cell lines were provided by J.L. Guan (University of Cincinnati, Cincinnati, Ohio, USA) (57, 58); and WT, LC3-DKO, and GABARAP-TKO MEF cell lines were provided by Osaka University (59). *Atg7*^+/+^ and *Atg7*^–/–^ MEFs were transfected with mNeonGreen-tagged TFEB. MEFs were grown in high-glucose DMEM (Nacalai Tesque, 08458-45) with 10% FBS and 1% penicillin/streptomycin. Sodium PA (MilliporeSigma, P9767) was dissolved in 100 mM NaOH and vigorously mixed with BSA (MilliporeSigma, A8806) at a molar ratio of 6.6:1. BSA and PA-bound BSA were added to the culture media at 0.125% and 0.125 mM, respectively, unless stated otherwise. PTECs were treated with 0.25 μM Torin 1 (Tocris, 4247) as a control. For amino acid starvation, PTECs were incubated for 60 minutes in HBSS (Thermo Fisher Scientific, 14025092). For amino acid refeeding, cells were restimulated for 30 minutes with 1× water-solubilized mix of essential (Thermo Fisher Scientific, 11130051) and nonessential (Thermo Fisher Scientific, 11140050) amino acids dissolved in HBSS (pH adjusted to 7.2–7.4 with NaOH) supplemented with 2 mM l-glutamine (Thermo Fisher Scientific, 25030149). To assess autophagic activity, PTECs were treated with 200 nM of bafilomycin A_1_ (Cayman Chemical, 11038) for 2 hours at 37°C before harvest. To evaluate ceramide toxicity, PTECs were treated with C_2_-ceramide (MilliporeSigma, A7191) for 12 hours.

### RNA-Seq analysis and bioinformatics.

PTECs were treated with either BSA or PA-bound BSA for 6 hours before harvest for RNA isolation. RNA-Seq was conducted by the Center of Medical Innovation and Translational Research, Osaka University, and by Macrogen Japan. The Illumina software package bcl2fastq was used for base calling. The raw reads were mapped to the house mouse reference genome sequence GRCm38 using TopHat (ver. 2.1.1) and Bowtie2 (ver. 2.3.4.1). Differential expression analysis was performed using the edgeR package (ver. 3.20.9). GSEA was conducted using the clusterProfiler package (ver. 3.12.0) in R (60). The fold change (FC) of gene expression between PA- and BSA-treated groups was calculated, and the gene list was generated according to the change of |log_2_FC|. KEGG pathway enrichment analyses were conducted using the gseKEGG function in the clusterProfiler package. An adjusted *P* < 0.05 was set as the cutoff criterion. A heatmap analysis was conducted using Morpheus software. RNA-Seq data were deposited in the Gene Expression Omnibus database (GSE191216) of the National Center for Biotechnology Information. We defined differentially expressed genes (DEGs) according to both an adjusted *P* < 0.05 and |log_2_FC| > 0.4. The number of DEGs was approximately 1,000. To identify TFs with enriched binding within 1 kb from the TSSs of genes differentially expressed by PA treatment, we performed ChIP-Atlas enrichment analysis of our RNA-Seq data and compared the results with published ChIP-Atlas data sets (http://chip-atlas.org/) (21). We used published data sets of *Homo* *sapiens* (GRCh38), because there are no published TFEB ChIP-Seq data of *Mus* *musculus*.

### Mice.

GFP-MAP1LC3 transgenic mice (gifted by N. Mizushima, University of Tokyo, Tokyo, Japan), *Tfeb^fl/fl^* KAP mice, and *Atg5^fl/fl^* KAP mice (gifted by N. Mizushima) on a C57BL/6 background have been described previously (12, 30, 61). Tamoxifen-inducible PTEC-specific *Tfeb*-deficient mice on a C57BL/6 background were generated by crossing mice bearing a *Tfeb*-floxed allele with N-myc downstream-regulated gene 1–CreERT2 (NDRG1-CreERT2) (provided by Kyoto University Graduate School of Medicine, Kyoto, Japan) mice (62, 63). Eight-week-old male mice were fed an ND (12.8% of kcal from fat: 5% fat, 23% protein, and 55% carbohydrate; Oriental Yeast, OYC2103800) or HFD (62.2% of kcal from fat: 35% fat, 23% protein, and 25% carbohydrate; Oriental Yeast, OYC2900100) for 2 or 10 months (Supplemental Table 4). To induce *Tfeb* deletion, tamoxifen (MilliporeSigma, T5648; 0.1 mg/g body weight) dissolved in corn oil (MilliporeSigma, C8267) was injected at a concentration of 10 mg/mL 3 days a week. *Tfeb^fl/fl^* NDRG1-CreERT2 mice were used only for the experiment validating exacerbated MLB accumulation due to *Tfeb* deficiency, because the use of corn oil to dissolve tamoxifen can modify metabolic profiles. To investigate the effect of TFEB activation, the following were performed during HFD treatment: intraperitoneal administration of saline or trehalose (MilliporeSigma, T9531; dissolved in saline, 2 mg/g body weight, 3 days a week) and oral administration of water or resveratrol (MilliporeSigma, R5010; dissolved in drinking water, 25 mg/L) in light-protected bottles. In an experiment assessing autophagic flux in vivo, chloroquine (MilliporeSigma, C6628; 50 mg/g body weight) was injected intraperitoneally 6 hours before euthanasia. Kidney IR and assessment of tubular injury were described previously (12, 64).

### Antibodies.

We used the following antibodies: LRP2/MEGALIN (gifted by T. Michigami, Department of Bone and Mineral Research, Osaka Medical Center and Research Institute for Maternal and Child Health, Osaka, Japan), TFEB (Bethyl, A303-673A, for mouse samples; Cell Signaling Technology, 37785, for human samples), phospho-TFEB (Ser211; Cell Signaling Technology, 37681), Lamin A/C (Cell Signaling Technology, 2032), GAPDH (GeneTex, GTX100118), S6RP (Cell Signaling Technology, 2217), phospho-S6RP (Ser235/236; Cell Signaling Technology, 2211), 4E-BP1 (Cell Signaling Technology, 9644), phospho-4E-BP1 (Thr37/46; Cell Signaling Technology, 2855), MTOR (Cell Signaling Technology, 2983), ACTB (MilliporeSigma, A5316), HA (MilliporeSigma, 11867423001), FLCN (Cell Signaling Technology, 3697), GABARAP (MBL, PM037), LAMP1 (BD Biosciences, 553792 for mouse samples; BD Biosciences, 555798 for human samples), CTSD (Santa Cruz Biotechnology, sc-6486), TMEM55B (Proteintech, 23992-1-AP), MAP1LC3B (Cell Signaling Technology, 2775), COL1A1 (Abcam, ab34710), F4/80 (Bio-Rad, MCA497), GALECTIN-3 (Santa Cruz Biotechnology, sc-23938), SQSTM1/p62 (PROGEN, GP62-C), biotinylated secondary antibodies (Vector Laboratories, BA-1000, BA-2001, BA-4000, and BA-7000), horseradish peroxidase–conjugated secondary antibodies (DAKO, P0447, P0448, P0449, and P0450), and Alexa Fluor–conjugated secondary antibodies (Thermo Fisher Scientific, A21206, A21208, A21434, and A31572).

### Histological analysis.

Histological analysis was performed as described previously, with modifications (16). The following were also performed as described previously: antigen retrieval on paraffin-embedded sections; double staining for LAMP1 and LRP2; electron microscopy analysis; toluidine blue, Nile red, and TUNEL staining; counting of GFP-MAP1LC3 dots; and assessment of kidney injury (10, 16, 64). TFEB activity was evaluated by counting the number of PTECs exhibiting TFEB nuclear translocation among total DAPI^+^ PTECs. For evaluation of PAS-stained sections, tubular vacuolation was graded semiquantitatively from 0 to 10 according to the percentage of vacuolated tubules. At least 10 high-power fields in each kidney were reviewed by 2 nephrologists in a blinded manner. For electron microscopy analysis, the numbers of MLBs, autophagosomes, and autolysosomes were counted. Basolateral MLBs were defined if they were located outside the line connecting the nuclei. At least 20 PTECs in each kidney were analyzed by 2 nephrologists in a blinded manner.

### Electron microscopy of urine pellets.

A total of 200 μL of urine was collected from both nonobese and obese *Tfeb^fl/fl^* mice and centrifuged at 1,000*g* for 10 minutes to remove cellular debris, then subsequently centrifuged at 20,000*g* for 30 minutes to obtain pellets (65). Pellets were fixed in 2% glutaraldehyde and observed using a Hitachi H-7650 transmission electron microscope.

### Lipidomics.

Urine was collected from nonobese and obese mice and centrifuged at 1,000*g* for 10 minutes to remove cellular debris. Lysosome-enriched subcellular fractions were isolated from kidneys using a modified version of a method described previously (66). Kidneys were homogenized with pestles in 1 mL of subcellular fractionation buffer (HEPES 20 mM, sucrose 250 mM, KCl 10 mM, MgCl_2_ 1.5 mM, EDTA 1 mM, EGTA 1 mM, dithiothreitol 8 mM, pH adjusted to 7.5 with NaOH). Debris and nuclei were pelleted at 750*g* for 12 minutes. The supernatant was centrifuged at 10,000*g* for 35 minutes to pellet the lysosome-enriched fraction. The pellet was washed once with subcellular fractionation buffer. Lipid extraction from urine and the lysosome-enriched fraction was performed using the Bligh and Dyer method with minor modifications (67). BMP, phosphatidylcholine, phosphatidylethanolamine, phosphatidylglycerol, phosphatidylinositol, phosphatidylserine, lysophosphatidylcholine, lysophosphatidylethanolamine, monoacylglycerol, diacylglycerol, triacylglycerol, cholesterol, ceramide, hexose ceramide, lactosylceramide, and sphingomyelin were analyzed by supercritical fluid chromatography (SFC) (Nexera UC system, Shimadzu; equipped with an ACQUITY UPC^2^ Torus diethylamine [DEA] column: 3.0 mm inner diameter [i.d.] × 100 mm, 1.7 μm particle size, Waters) and triple quadrupole mass spectrometry (TQMS; LCMS-8060, Shimadzu) (DEA-SFC/MS/MS) in multiple reaction monitoring (MRM) mode (68). Fatty acids and cholesterylester were analyzed using an SFC (Shimadzu) with an ACQUITY UPC^2^ HSS C18 SB column (3.0 mm i.d. × 100 mm, 1.8 μm particle size, Waters) coupled with a TQMS (Shimadzu) (C18-SFC/MS/MS) in MRM mode (69). The amount of each lipid species was normalized either to the urine creatinine concentration, measured using a QuantiChrom Creatinine Assay Kit (DICT-500) (BioAssay Systems), or to kidney weight.

### Nuclear and cytosolic/cytoplasmic fractionation.

Kidney nuclear and cytosolic fractionation were performed as described previously (26). Kidneys were homogenized with pestles in cytosol isolation buffer (250 mM sucrose, 20 mM HEPES, 10 mM KCl, 1.5 mM MgCl_2_, 1 mM EDTA, and 1 mM EGTA) supplemented with protease and phosphatase inhibitors, followed by centrifugation at 1,000*g* for 10 minutes at 4°C to pellet the nuclei. The supernatant (cytosolic fraction) was recentrifuged twice at 16,000*g* for 20 minutes at 4°C to pellet the mitochondria and debris. Nuclear pellets were washed with PBS and centrifuged at 800*g* for 10 minutes 3 times, resuspended in nuclear lysis buffer (1.5 mM MgCl_2_, 0.2 mM EDTA, 20 mM HEPES, 0.5 M NaCl, 20% glycerol, 1% Triton X-100) supplemented with protease and phosphatase inhibitors, incubated on ice for 30 minutes (vortexed every 10 minutes), and then sonicated. Finally, samples were centrifuged at 16,000 *g* for 15 minutes, and the supernatant containing the enriched nuclear fraction was collected. For nuclear and cytoplasmic fractionation in vitro, PTECs were lysed in PBS containing 0.1% NP-40 and protease and phosphatase inhibitors, then centrifuged at 9,100*g* for 10 seconds. The pellet (nuclear fraction) was washed once with lysis buffer. The supernatant (cytoplasmic fraction) and nuclear fraction were lysed in SDS sample buffer.

### Assessment of TFEB nuclear localization.

TFEB was considered translocated to the nucleus only when nuclear TFEB was clearly detected. The percentage of PTECs exhibiting TFEB nuclear translocation was determined by counting more than 100 cells from more than 5 fields (70).

### Fluorescent fatty acid pulse-chase assay.

A fluorescent fatty acid pulse-chase assay was performed as described previously, with slight modification (71). PTECs were incubated with low-glucose DMEM and 5% FBS containing 2 μM β-BODIPY FL HPC (Thermo Fisher Scientific, D3792) for 16 hours, then washed 3 times with DMEM with 5% FBS, incubated for 1 hour to allow the fluorescent lipids to incorporate into cellular membranes, and chased for 6 hours under BSA or PA treatment. PTECs were then stained with 50 nM LysoTracker Red DND-99 (Thermo Fisher Scientific, L7528) at 37°C for 30 minutes before imaging. FL-HPC–positive and LysoTracker-negative dots outside the cells were counted as released phospholipids. In addition, to assess the clearance of lysosomal phospholipid accumulation, we observed PA-treated PTECs for 12 or 24 hours after PA washout. The number of dots indicating staining for both phospholipids and lysosomes was determined using the ImageJ (NIH) “analyze particles” function.

### β-Hexosaminidase activity assay.

A β-hexosaminidase activity assay was performed largely according to previous reports (72, 73). PTECs were cultured in 12-well plates to confluence, followed by treatment with BSA or PA. Before measurement, we washed the PTECs with PBS twice and then added 300 μL of PBS containing 1.2 mM CaCl_2_. After 10 minutes, culture supernatants were collected. To determine the total cellular content of β-hexosaminidase, PTECs were incubated in 300 μL of 0.5% Triton X-100 for 15 minutes at 37°C. For each sample, 175 μL was incubated for 15 minutes at 37°C with 25 μL of 4 mM 4-methyl-umbellyferyl-*N*-acetyl-β-d glucosaminide in 20 mM sodium citrate phosphate buffer (pH 4.5). The reaction was stopped by the addition of 50 μL of 2 M Na_2_CO_3_ and 1.1 M glycine, and the fluorescence was measured in an SH9000 spectrofluorometer (Corona Electric) using an excitation wavelength of 365 nm and an emission wavelength of 450 nm. The β-hexosaminidase activity (supernatant/total cellular activity) was normalized by the value of untreated control wild-type or *Tfeb*-deficient PTECs (cultured in DMEM containing 5% FBS).

### LDH activity assay.

LDH activity assays were performed using the Cytotoxicity Detection KitPLUS (LDH) (Roche Diagnostics). LDH activity (supernatant/total cellular activity) was normalized by the value of untreated control wild-type or *Tfeb*-deficient PTECs (cultured in DMEM containing 5% FBS).

### Staining of LAMP1 on the plasma membrane.

Staining of surface LAMP1 was performed largely according to a previous study (74). PTECs were treated with BSA or PA, followed by incubation with PBS containing 1.2 mM CaCl_2_ for 10 minutes. Thereafter, PTECs were transferred on ice, then incubated with anti–rat LAMP1-1D4B (Thermo Fisher Scientific,14-1071-82) for 30 minutes. PTECs were washed in cold PBS and fixed in 2% paraformaldehyde. PTECs were further incubated at room temperature with Alexa Fluor–conjugated secondary antibodies (see *Antibodies*) for 30 minutes and with DAPI for 3 minutes.

### Cell proliferation assay.

MTS assays were performed using the CellTiter 96 Aqueous One Solution Cell Proliferation Assay (Promega, G3582) in a 96-well plate. Each experiment was repeated at least 3 times. All values are standardized to untreated control cells (cultured in DMEM containing 5% FBS).

### Quantitative reverse transcription PCR and Western blot analysis.

Quantitative reverse transcription PCR and Western blot analyses were performed as described previously (75). The sequences of the primers used were as follows: *Tmem55b*-F, 5′ CCTCACTGCAGAAAAGTGTCA 3′; *Tmem55b*-R, 5′ TGACTGCCAAGAGTAAACCAAG 3′; *Gapdh*-F, 5′ AACTTTGGCATTGTGGAAGG 3′; and *Gapdh*-R, 5′ ACACATTGGGGGTAGGAACA 3′.

### Kidney biopsy specimens.

Human kidney specimens were obtained from patients who had undergone renal biopsy at the Osaka University Hospital. The degree of vacuole formation was defined by the number of vacuoles divided by the length of the cortex (mm) on PAS staining. The percentage of PTECs exhibiting TFEB nuclear translocation was assessed in at least 5 randomly selected high-power fields (original magnification, ×400). In all quantitative analyses of histological stains, at least 5 high-power fields were reviewed by 3 nephrologists in a blinded manner.

### Statistics.

All results are presented as bar graphs showing means ± SEM. Statistical analyses were conducted using JMP software (SAS Institute). Multiple-group comparisons were performed using 1-way ANOVA with posttesting using the Tukey-Kramer test. In Figure 3A and Supplemental Figure 6C, 1-way ANOVA and then Dunnett’s test were used to detect intergroup differences. The difference between any 2 experimental values was assessed using the 2-tailed Student’s *t* test when appropriate. Statistical significance was defined as *P* < 0.05. Relationships were examined using Pearson’s correlation and the corresponding *P* values.

### Study approval.

All animal experiments were approved by the Animal Research Committee of Osaka University and conformed to the Japanese Animal Protection and Management Law (No. 25). All patients who had undergone renal biopsy had provided written informed consent.

## Author contributions

JN, S Minami, and T Yamamoto designed the study; JN and T Yamamoto carried out most experiments, analyzed and interpreted the data, and drafted the manuscript; YT interpreted data and edited/revised the manuscript; TNH, S Minami, AT, JM, SS, HY, S Maeda, S Matsui, and IM helped with histologic analyses; TH helped with statistical analyses; MT, MG, Y Izumi, and TB performed lipidomic analysis; MS, M Yamamoto, TM, FN, and M Yanagita provided cell lines or mice and commented on the manuscript; SN, T Yoshimori, AB, and Y Isaka provided intellectual input; and all authors contributed to the discussions and approved the final version of the manuscript.

## Supplementary Material

Supplemental data

## Version 1 01/17/2023

In-Press Preview

## Version 2 02/22/2023

Electronic publication
