# Supplementary material for: TFEB-mediated lysosomal exocytosis alleviates high-fat diet–induced lipotoxicity in the kidney
Source: JCI Insight. 2023 Feb 22;8(4):e162498. doi: 10.1172/jci.insight.162498 (PMC9977505; doi:10.1172/jci.insight.162498)
Supplement: Supplemental data [file jciinsight-8-162498-s056.pdf]

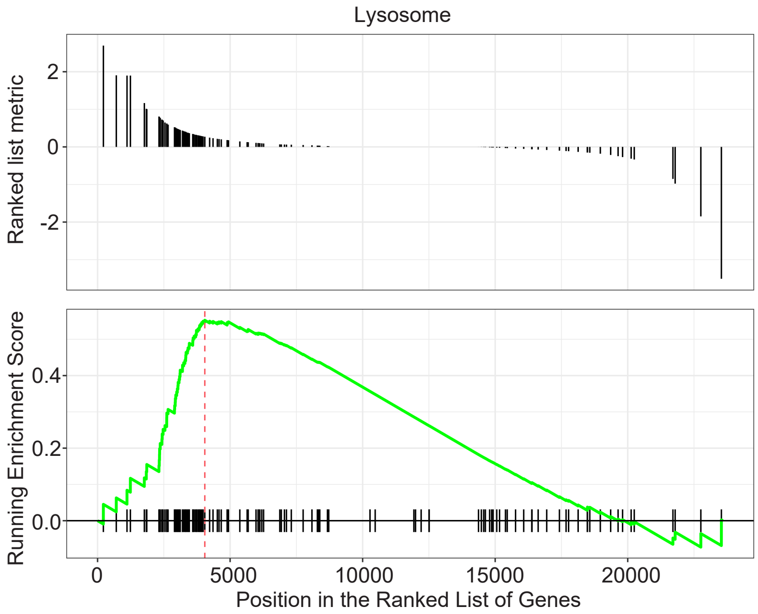

Supplemental Figure 1. The "Lysosome" KEGG pathway is globally upregulated by PA treatment. We performed RNA-seq transcriptomic analysis using cultured PTECs treated with either BSA or PA-bound BSA for 6 hours (n = 3). GSEA-based KEGG-enrichment plots show that the "Lysosome" pathway is upregulated by PA treatment. The running enrichment score is plotted in green as a function of the position in the ranked list of genes.

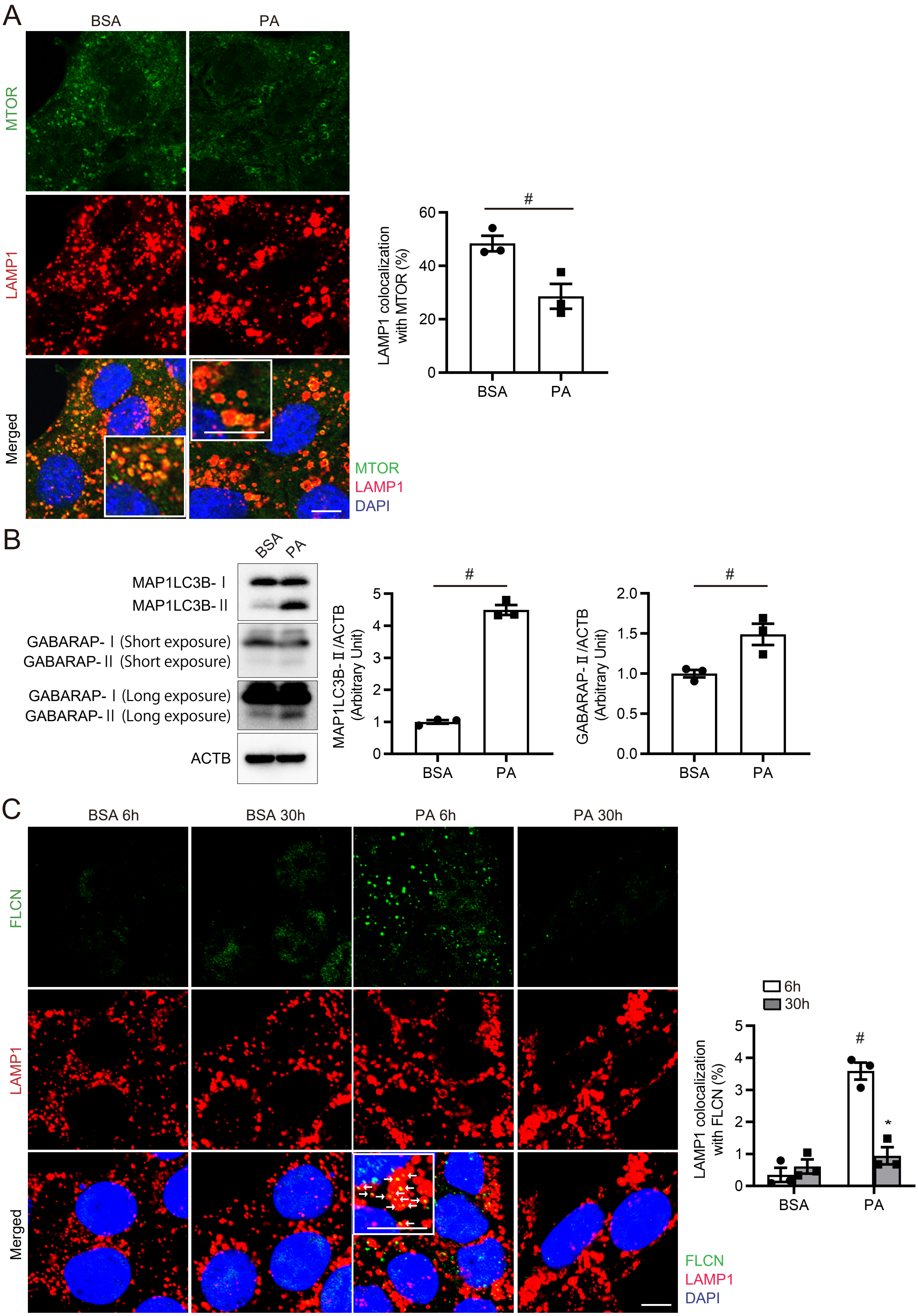
A
BSA
PA
MTOR
LAMP1
Merged
MTOR
LAMP1
DAPI
LAMP1 colocalization with MTOR (%)
60
40
20
0
#
BSA
PA
B
BSA
PA
MAP1LC3B- I
MAP1LC3B- II
GABARAP- I (Short exposure)
GABARAP- II (Short exposure)
GABARAP- I (Long exposure)
GABARAP- II (Long exposure)
ACTB
MAP1LC3B- II /ACTB (Arbitrary Unit)
5
4
3
2
1
0
#
BSA
PA
GABARAP- II /ACTB (Arbitrary Unit)
2.0
1.5
1.0
0.5
0.0
#
BSA
PA
C
BSA 6h
BSA 30h
PA 6h
PA 30h
FLCN
LAMP1
Merged
FLCN
LAMP1
DAPI
6h
30h
LAMP1 colocalization with FLCN (%)
5
4
3
2
1
0
#
*
BSA
PA

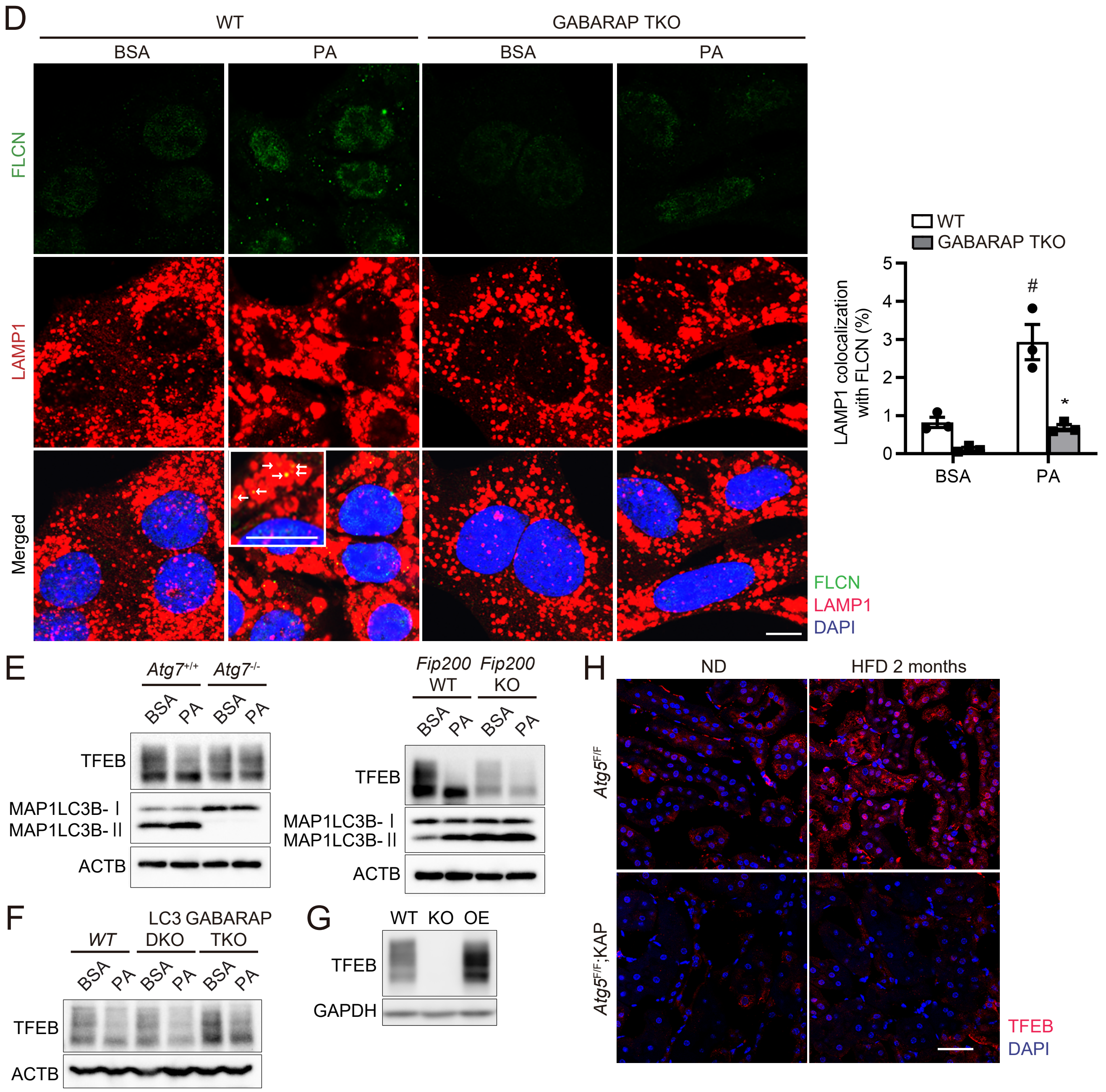

Supplemental Figure 2. PA-induced TFEB activation is ATG conjugation system dependent. (**A**) Immunofluorescence images of MTOR and LAMP1 in cultured PTECs after BSA or PA treatment for 6 hours (n = 3). The total number of LAMP1 dots and the number of LAMP1 dots that colocalized with MTOR were quantified. Data are presented as the percentage of LAMP1 dots that colocalized with MTOR. (**B**) Western blot images of MAP1LC3B, and GABARAP in cultured PTECs after BSA or PA treatment for 6 hours (n = 3). Densitometric analysis was performed. (**C** and **D**) Immunofluorescence images of FLCN and LAMP1 after BSA (0.125% (**C**) or 0.25% (**D**)) or PA (0.125 mM (**C**) or 0.25 mM (**D**)) treatment for 6 or 30 hours in cultured PTECs (**C**), or for 6 hours in wild-type and *Gabarap*, *Gabarapl1*, and *Gabarapl2* triple knockout (GABARAP TKO) MEFs (**D**) (n = 3). The total number of LAMP1 dots and the number of LAMP1 dots that colocalized with FLCN were quantified. Data are presented as the percentage of LAMP1 dots that colocalized with FLCN. (**E** and **F**) Western blot images of TFEB and MAP1LC3B in the following cells after BSA (0.25%) or PA (0.25mM) treatment for 6 hours: wild-type and *Atg7*-deficient MEFs or wild-type and *Fip200*-deficient MEFs (**E**); and wild-type, *Map1lc3a* and *Map1lc3b* double knockout (LC3 DKO), and GABARAP TKO MEFs (**F**). (**G**) Western blot images of TFEB in wild-type, *Tfeb*-deficient, and *Tfeb*-overexpressing PTECs. (**H**) Immunostaining images of TFEB in the kidney cortical regions of nonobese and obese *Atg5*$^{F/F}$ or *Atg5*$^{F/F}$;KAP mice (n = 3–4). Bars: 10 μm (**A**, **C** and **D**) and 40 μm (**H**). Data are provided as means ± SE. Statistically significant differences: $^{*}P < 0.05$ versus corresponding treatment for 6 hours or wild-type PTECs; $^{\#}P < 0.05$ versus BSA-treated PTECs (**A** and **B**, two-tailed Student' s *t*-test; **C** and **D**, one-way ANOVA followed by the Tukey-Kramer test). All images are representative of multiple experiments. Arrows indicate merged dots. n.s., not significant; WT, wild-type PTEC; KO, *Tfeb*-deficient PTEC; OE, *Tfeb*-overexpressing PTEC.

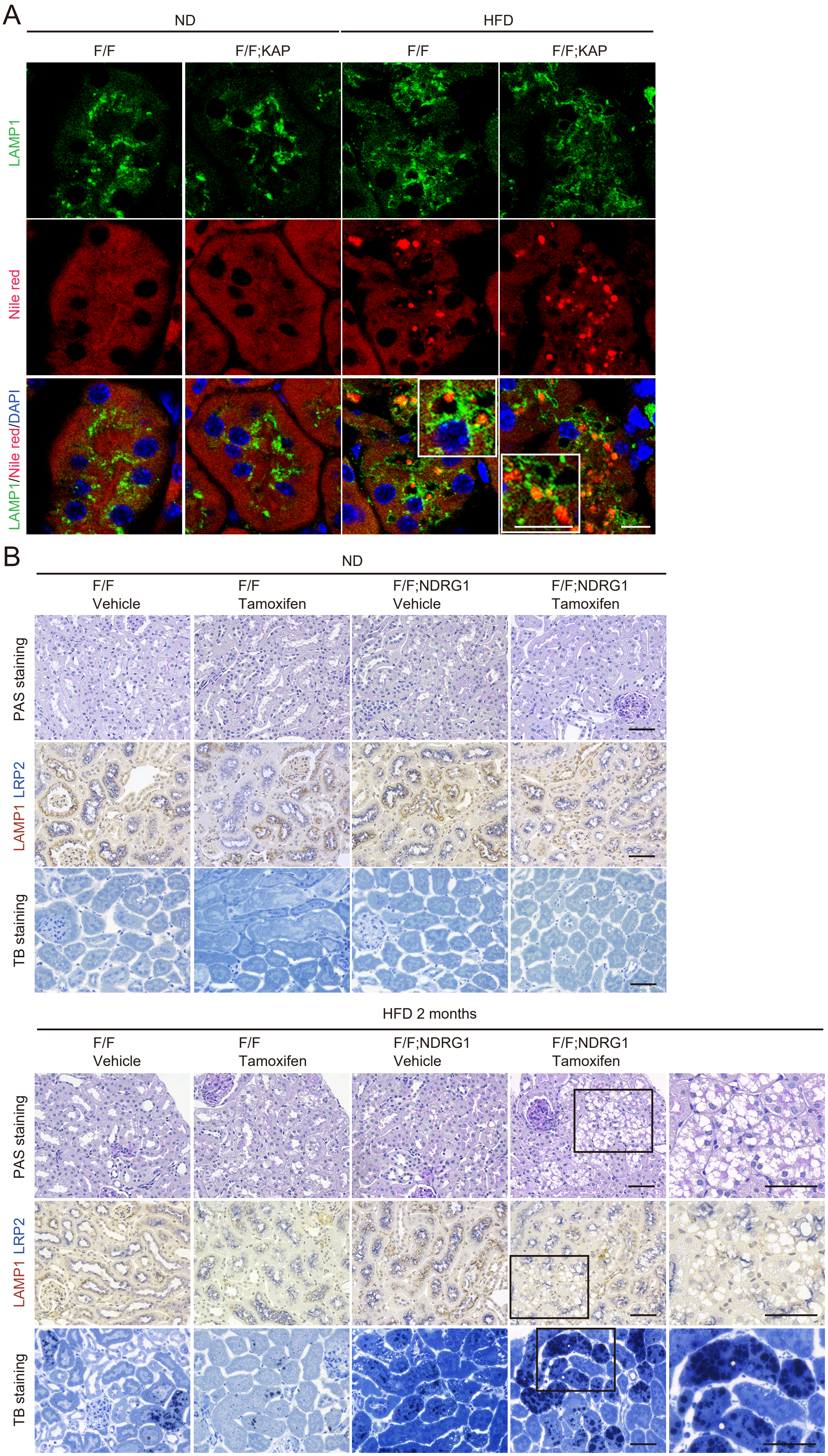
A
ND
HFD
F/F
F/F;KAP
F/F
F/F;KAP
LAMP1
Nile red
LAMP1/Nile red/DAPI
B
ND
F/F Vehicle
F/F Tamoxifen
F/F;NDRG1 Vehicle
F/F;NDRG1 Tamoxifen
PAS staining
LAMP1 LRP2
TB staining
HFD 2 months
F/F Vehicle
F/F Tamoxifen
F/F;NDRG1 Vehicle
F/F;NDRG1 Tamoxifen
PAS staining
LAMP1 LRP2
TB staining

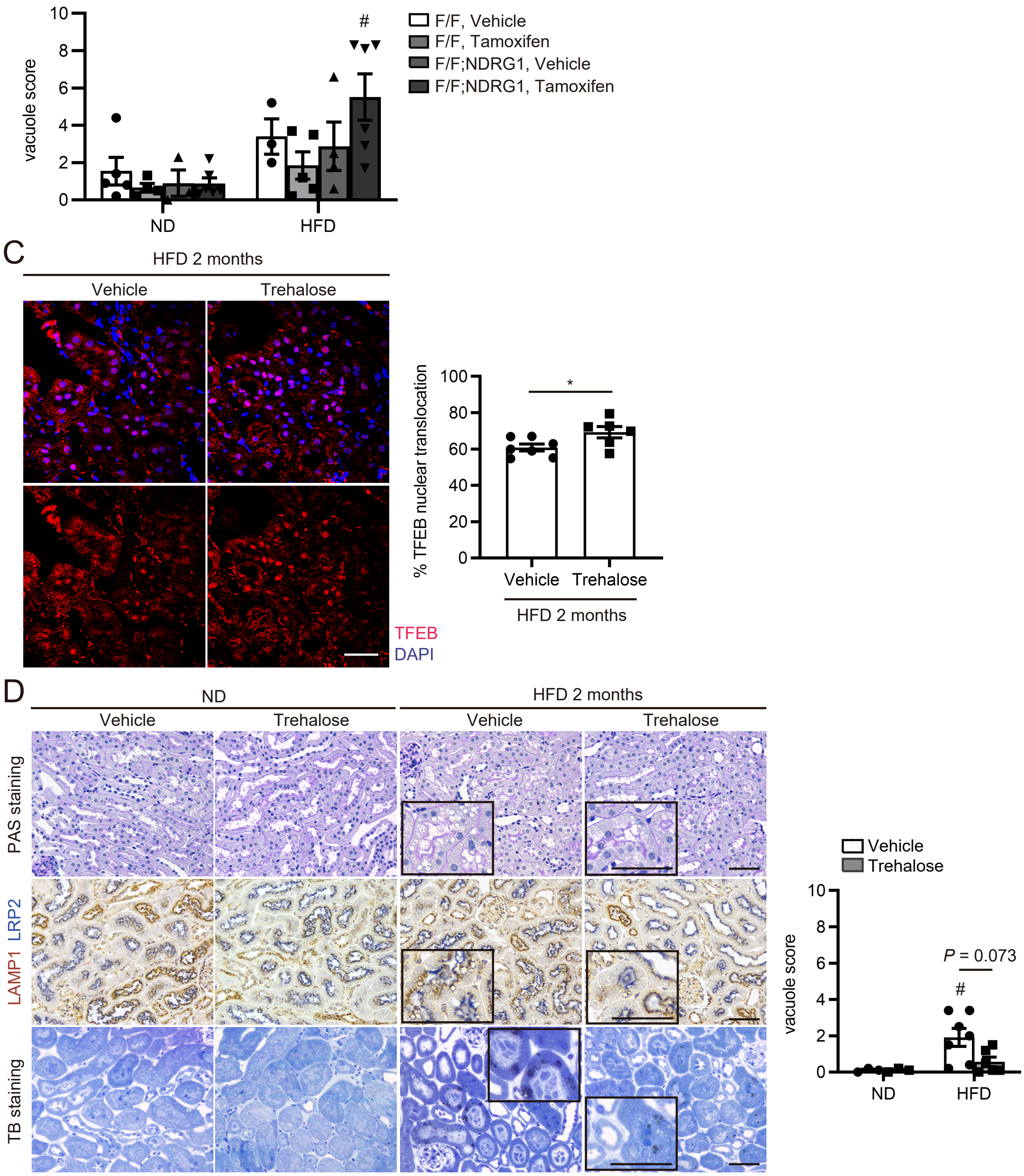
vacuole score
F/F, Vehicle
F/F, Tamoxifen
F/F;NDRG1, Vehicle
F/F;NDRG1, Tamoxifen
#
ND
HFD
C
HFD 2 months
Vehicle
Trehalose
TFEB
DAPI
% TFEB nuclear translocation
*
Vehicle
Trehalose
HFD 2 months
D
ND
HFD 2 months
Vehicle
Trehalose
Vehicle
Trehalose
PAS staining
LAMP1 LRP2
TB staining
Vehicle
Trehalose
vacuole score
P = 0.073
#
ND
HFD

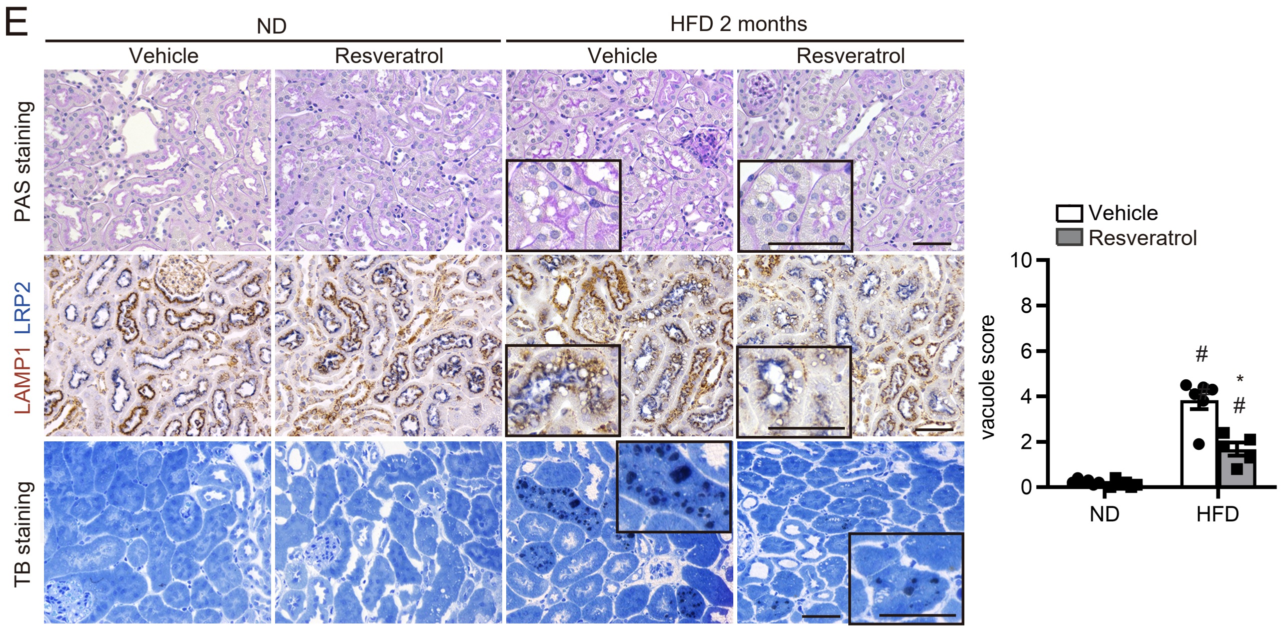

Supplemental Figure 3. TFEB reduces phospholipid accumulation in enlarged lysosomes in PTECs. The effect of TFEB on phospholipid accumulation in enlarged lysosomes in PTECs during lipid overload was investigated using *Tfeb*$^{F/F}$;KAP mice, *Tfeb*$^{F/F}$;NDRG1-CreERT2 mice (another type of PTEC-specific *Tfeb*-deficient mouse) and wild-type mice, the last of which were treated with trehalose or resveratrol, both of which are TFEB activators. (**A**) Immunostaining images of LAMP1 (green) and Nile red (red) in kidney cortical regions of nonobese and obese *Tfeb*$^{F/F}$ or *Tfeb*$^{F/F}$;KAP mice (n = 5–6). Magnified images are shown in the insets (original magnification, × 600). Sections were counterstained with DAPI. (**B**–**E**). *Tfeb*$^{F/F}$;NDRG1-CreERT2 mice and *Tfeb*$^{F/F}$ control mice (**B**) or vehicle- and trehalose-treated wild-type mice (**C** and **D**), and vehicle- and resveratrol-treated wild-type mice (**E**), all at 8 weeks of age, were fed a ND or HFD for 2 months. Images showing PAS staining, toluidine blue staining, and LAMP1 or TFEB immunostaining in kidney cortical regions (n = 3–6). Magnified images are shown in the insets (original magnification, × 400). Sections were immunostained for LRP2, a marker of proximal tubules (blue), and counterstained with hematoxylin (blue). The vacuole scores are shown. Bars: 10 μm (**A**), 40 μm (**C**), and 50 μm (**B**, **D**, and **E**). Values represent means ± SE. Statistically significant differences: $^{*}P < 0.05$ versus vehicle-treated mice; $^{\#}P < 0.05$ versus nonobese mice (**B**, **D** and **E**, one-way ANOVA followed by the Tukey-Kramer test; **C**, two-tailed Student' s *t*-test). All images are representative of multiple experiments. F/F, *Tfeb*$^{F/F}$ mice; F/F;KAP, *Tfeb*$^{F/F}$;KAP mice, F/F; NDRG1, *Tfeb*$^{F/F}$; NDRG1-CreERT2 mice.

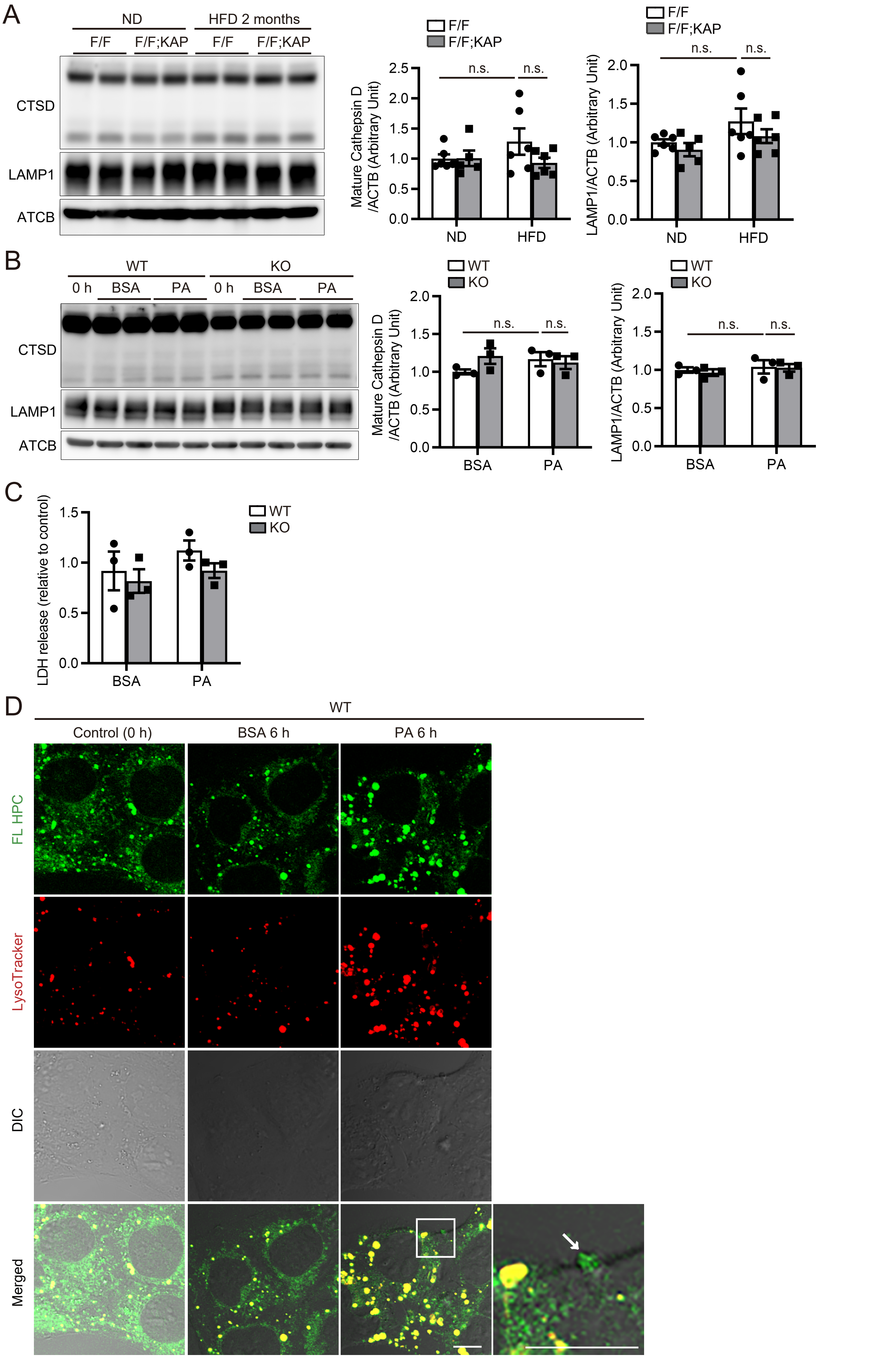
A
ND
HFD 2 months
F/F
F/F;KAP
F/F
F/F;KAP
CTSD
LAMP1
ATCB
F/F
F/F;KAP
n.s.
n.s.
Mature Cathepsin D /ACTB (Arbitrary Unit)
2.5
2.0
1.5
1.0
0.5
0.0
ND
HFD
F/F
F/F;KAP
n.s.
n.s.
LAMP1/ACTB (Arbitrary Unit)
2.0
1.5
1.0
0.5
0.0
ND
HFD
B
WT
KO
0 h
BSA
PA
0 h
BSA
PA
CTSD
LAMP1
ATCB
WT
KO
n.s.
n.s.
Mature Cathepsin D /ACTB (Arbitrary Unit)
2.0
1.5
1.0
0.5
0.0
BSA
PA
WT
KO
n.s.
n.s.
LAMP1/ACTB (Arbitrary Unit)
2.0
1.5
1.0
0.5
0.0
BSA
PA
C
LDH release (relative to control)
1.5
1.0
0.5
0.0
BSA
PA
WT
KO
D
WT
Control (0 h)
BSA 6 h
PA 6 h
FL HPC
LysoTracker
DIC
Merged

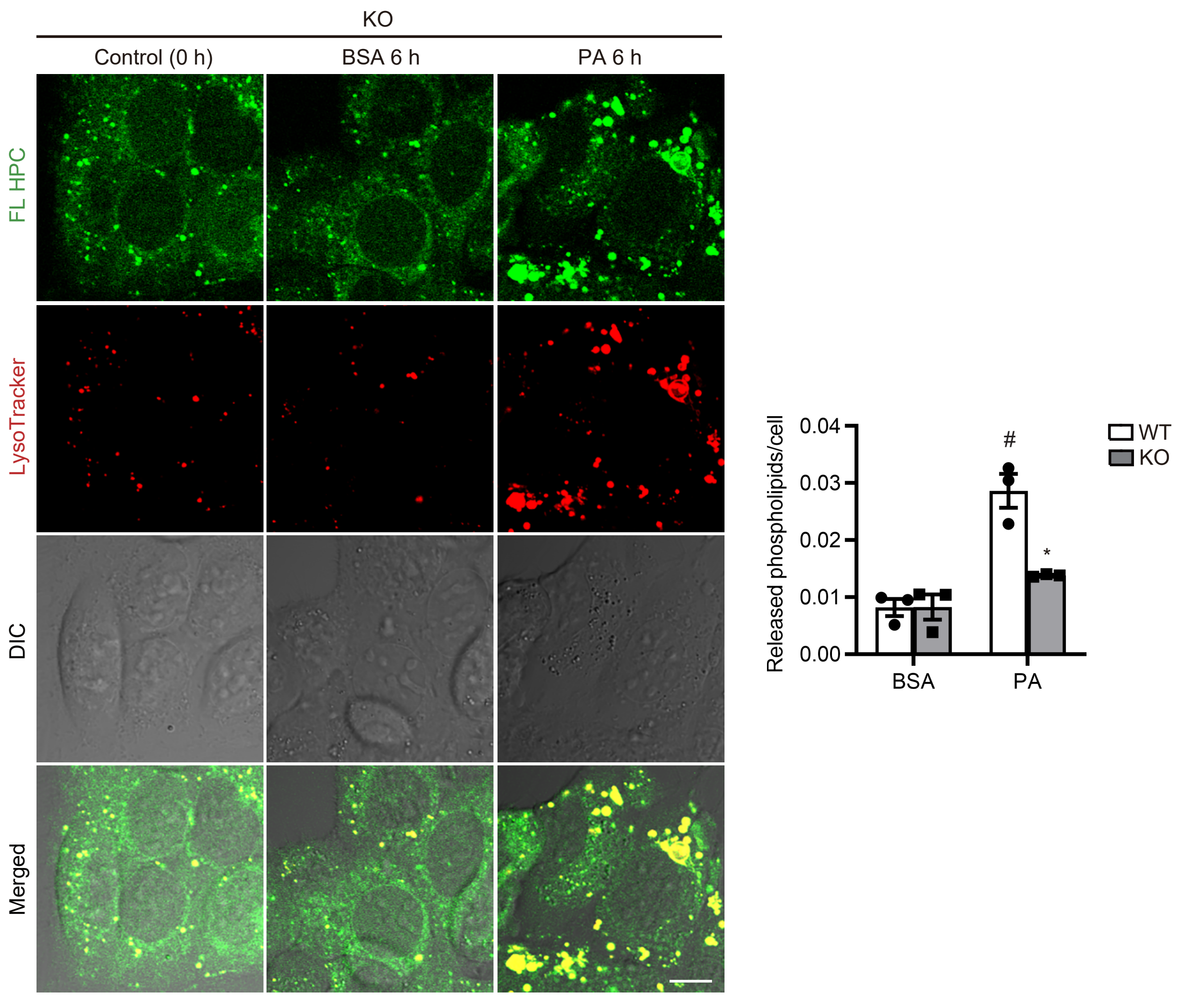

Supplemental Figure 4. TFEB is not involved in lysosomal biogenesis or proteolytic activity during lipid overload. Representative western blot images of CTSD and LAMP1 in whole kidney lysates of nonobese and obese *Tfeb*$^{F/F}$ or *Tfeb*$^{F/F}$;KAP mice (n = 5–6) (**A**), and cultured PTECs after BSA or PA treatment for 6 hours (n = 3) (**B**). (**C**) LDH activity in the culture supernatant of PTECs relative to the total activity after BSA or PA treatment for 6 hours (n = 3). (**D**) To investigate the trafficking of phospholipids, a fluorescent fatty acid pulse-chase assay was performed. FL HPC–loaded wild-type and *Tfeb*-deficient PTECs were chased after treatment with either 0.125% BSA or 0.125 mM PA for 6 hours, and the subcellular localization of FL HPC was determined by staining with LysoTracker Red. FL HPC–positive and Lysotracker-negative dots outside the cells were counted as released phospholipids (n = 3). Magnified images are shown in the insets (original magnification, × 600). Bars: 10 μm (**D**).Data are provided as means ± SE. Statistically significant differences: $^{*}P$ < 0.05 versus treatment-matched *Tfeb*$^{F/F}$ control littermates or wild-type PTECs; $^{\#}P$ < 0.05 versus nonobese mice or BSA-treated PTECs (**A-D**, one-way ANOVA followed by the Tukey-Kramer test). n.s., not significant; F/F, *Tfeb*$^{F/F}$ mice; F/F;KAP, *Tfeb*$^{F/F}$;KAP mice; WT, wild-type PTEC; KO, *Tfeb*-deficient PTEC.

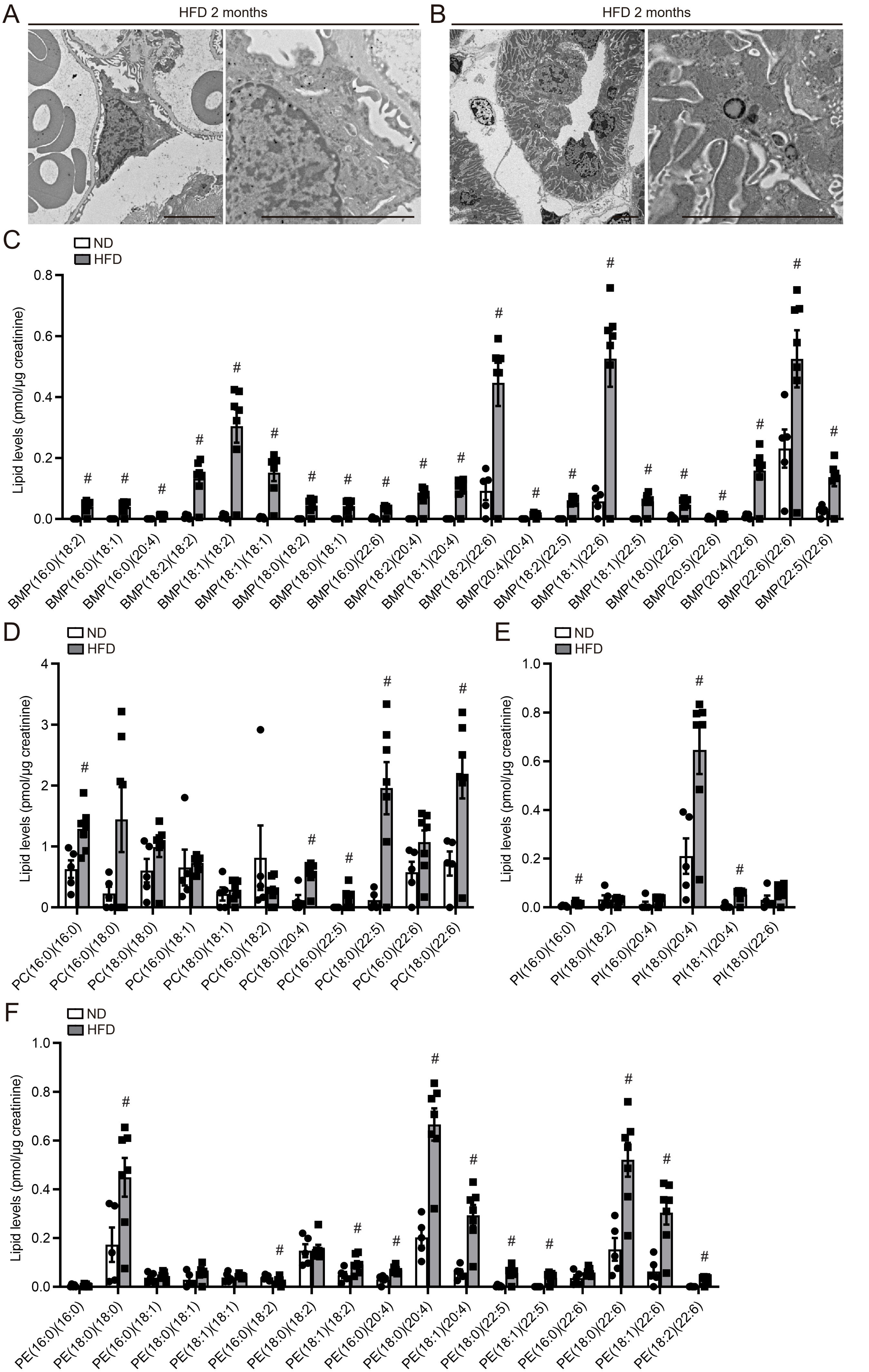
A
HFD 2 months
B
HFD 2 months
C
ND
HFD
Lipid levels (pmol/μg creatinine)
0.0
0.2
0.4
0.6
0.8
BMP(16:0)(18:2)
BMP(16:0)(18:1)
BMP(16:0)(20:4)
BMP(18:2)(18:2)
BMP(18:1)(18:2)
BMP(18:1)(18:1)
BMP(18:0)(18:2)
BMP(18:0)(18:1)
BMP(16:0)(22:6)
BMP(18:2)(20:4)
BMP(18:1)(20:4)
BMP(18:2)(22:6)
BMP(20:4)(20:4)
BMP(18:2)(22:5)
BMP(18:1)(22:6)
BMP(18:1)(22:5)
BMP(18:0)(22:6)
BMP(20:5)(22:6)
BMP(20:4)(22:6)
BMP(22:6)(22:6)
BMP(22:5)(22:6)
D
ND
HFD
Lipid levels (pmol/μg creatinine)
0
1
2
3
4
PC(16:0)(16:0)
PC(16:0)(18:0)
PC(18:0)(18:0)
PC(16:0)(18:1)
PC(18:0)(18:1)
PC(16:0)(18:2)
PC(18:0)(20:4)
PC(16:0)(22:5)
PC(18:0)(22:5)
PC(16:0)(22:6)
PC(18:0)(22:6)
E
ND
HFD
Lipid levels (pmol/μg creatinine)
0.0
0.2
0.4
0.6
0.8
1.0
PI(16:0)(16:0)
PI(18:0)(18:2)
PI(16:0)(20:4)
PI(18:0)(20:4)
PI(18:1)(20:4)
PI(18:0)(22:6)
F
ND
HFD
Lipid levels (pmol/μg creatinine)
0.0
0.2
0.4
0.6
0.8
1.0
PE(16:0)(16:0)
PE(18:0)(18:0)
PE(16:0)(18:1)
PE(18:0)(18:1)
PE(18:1)(18:1)
PE(16:0)(18:2)
PE(18:0)(18:2)
PE(18:1)(18:2)
PE(16:0)(20:4)
PE(18:0)(20:4)
PE(18:1)(20:4)
PE(18:0)(22:5)
PE(18:1)(22:5)
PE(16:0)(22:6)
PE(18:0)(22:6)
PE(18:1)(22:6)
PE(18:2)(22:6)

Supplemental Figure 5. Urinary levels of phospholipid species in obese mice. (**A** and **B**) Representative electron micrographs of the distal tubules (**A**) and podocytes (**B**) of obese *Tfeb*$^{F/F}$ mice (n = 3). (**C**–**F**) Urinary lipidomic profiles of nonobese and obese mice (n = 5 or 7 in each group). Data are presented as the amount of each phospholipid species normalized to the urine creatinine concentration. Bars: 5 μm (**A** and **B**). Values represent means ± SE. Statistically significant differences: $^{\#}P < 0.05$ versus nonobese mice (**C**-**F**, two-tailed Student' s *t*-test).. BMP, bis(monoacylglycerol) phosphate; PC, phosphatidylcholine; PI, phosphatidylinositol; PE, phosphatidylethanolamine.

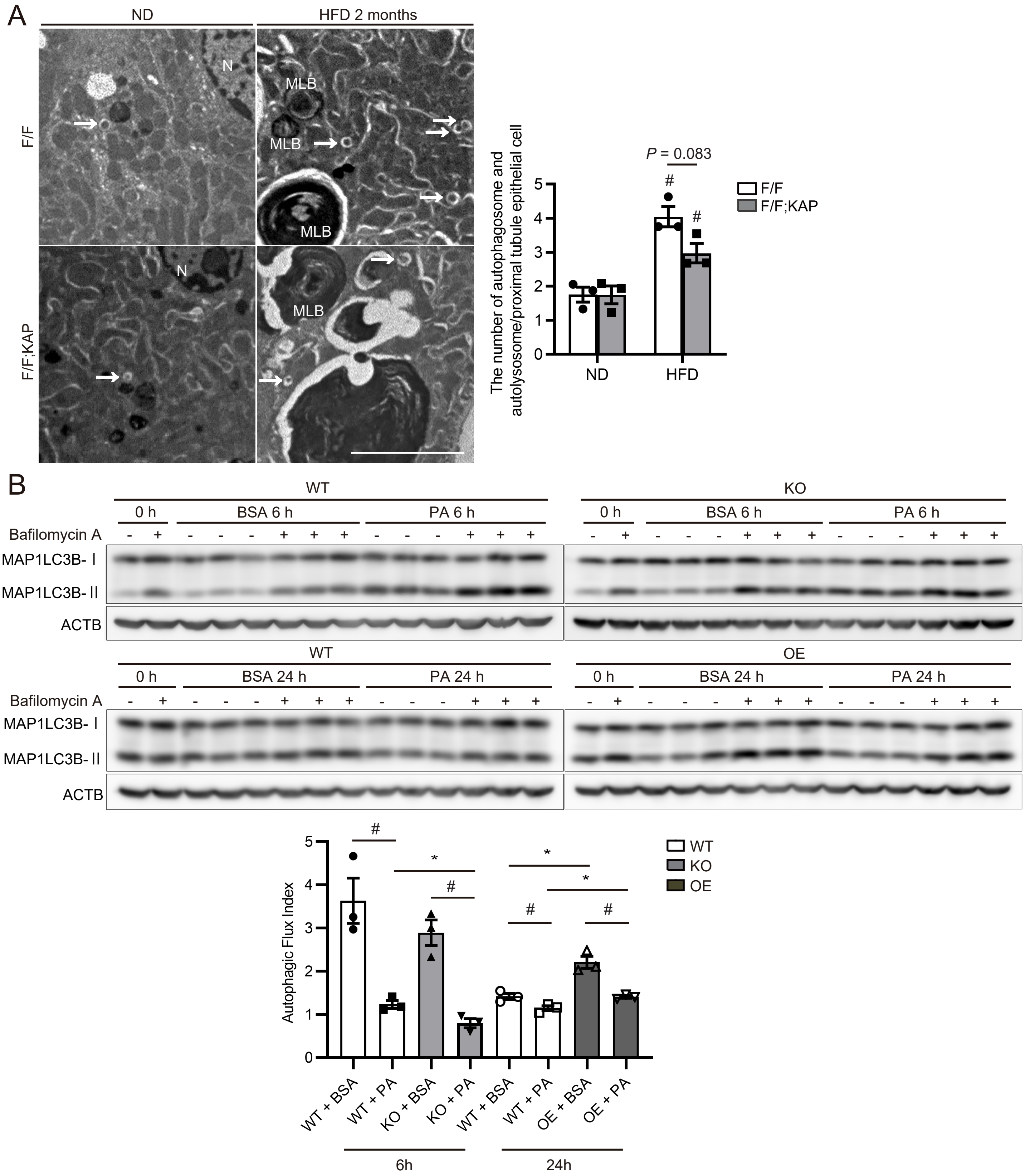
A
ND
HFD 2 months
F/F
F/F;KAP
N
MLB
The number of autophagosome and autolysosome/proximal tubule epithelial cell
P = 0.083
#
F/F
F/F;KAP
ND
HFD
B
WT
KO
0 h
BSA 6 h
PA 6 h
Bafilomycin A
MAP1LC3B- I
MAP1LC3B- II
ACTB
OE
BSA 24 h
PA 24 h
Autophagic Flux Index
WT
KO
OE
WT + BSA
WT + PA
KO + BSA
KO + PA
OE + BSA
OE + PA
6h
24h

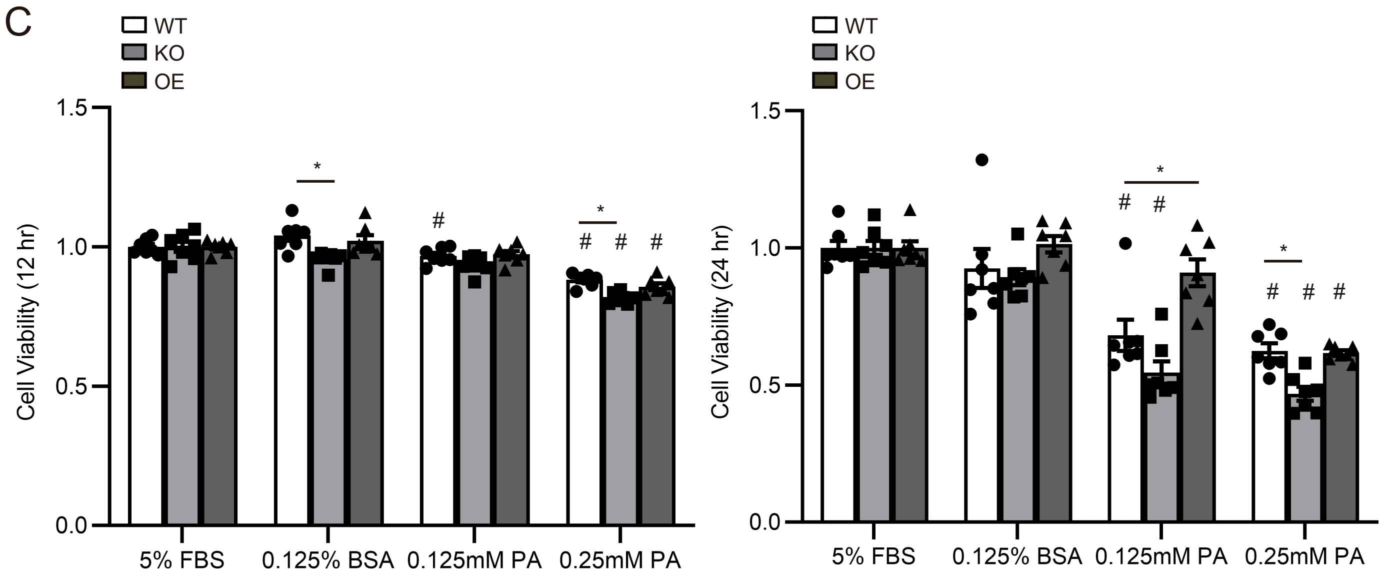

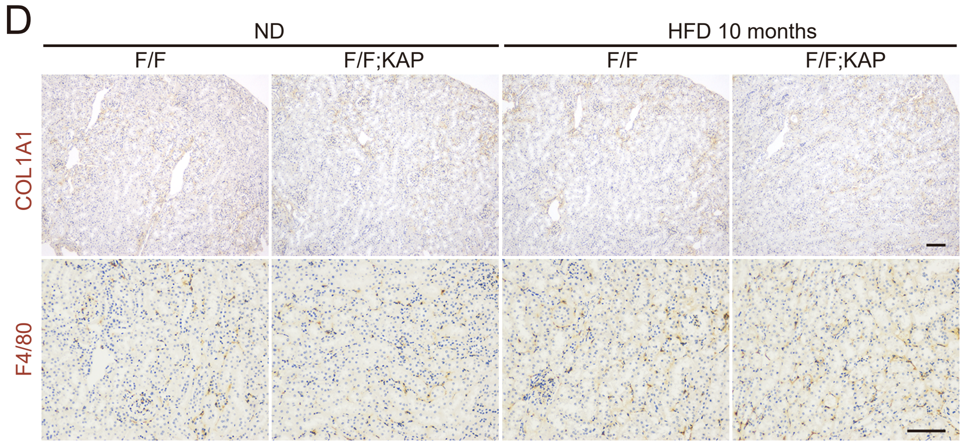

Supplemental Figure 6. TFEB alleviates impairment in autophagic flux and cell viability following PA overload. (**A**) Electron micrograph images of the kidneys of nonobese and obese *Tfeb*$^{F/F}$ or *Tfeb*$^{F/F}$;KAP mice. The number of autophagosomes or autolysosomes per proximal tubule epithelial cell under each condition was counted in at least 20 PTECs (n = 3). (**B** and **C**) To investigate the role of TFEB in autophagic flux and cell viability, wild-type, *Tfeb*-deficient, and *Tfeb*-overexpressing PTECs were treated with BSA or PA for the indicated periods. (**B**) Western blot images of MAP1LC3B (n = 3). The autophagic flux index is defined as the ratio of the MAP1LC3B-II level in the presence of bafilomycin A1 to that in the absence of bafilomycin A1. (**C**) Cell survival of PTECs treated with the indicated concentrations of FBS, BSA, or PA (n = 7). (**D**) *Tfeb*$^{F/F}$ or *Tfeb*$^{F/F}$;KAP mice were fed with a ND or HFD for 10 months. Immunostaining images of COL1A1 and F4/80 in the kidney cortical regions (n = 6–9). Sections were counterstained with hematoxylin (blue). Bars: 5 μm (**A**) and 100 μm (**D**). Data are provided as means ± SE. Statistically significant differences: $^{*}P$ < 0.05 versus wild-type PTECs with the same treatment; $^{\#}P$ < 0.05 versus nonobese mice or versus BSA-treated cells (**A** and **C**, one-way ANOVA followed by the Tukey-Kramer test (**A**) or Dunnett' s test (**C**); **B**, two-tailed Student' s *t*-test). All images are representative of multiple experiments. Arrows indicate autophagosomes. MLB, multilamellar body; N, nucleus; F/F, *Tfeb*$^{F/F}$ mice; F/F;KAP, *Tfeb*$^{F/F}$;KAP mice; WT, wild-type PTEC; KO, *Tfeb*-deficient PTEC; OE, *Tfeb*-overexpressing PTEC.

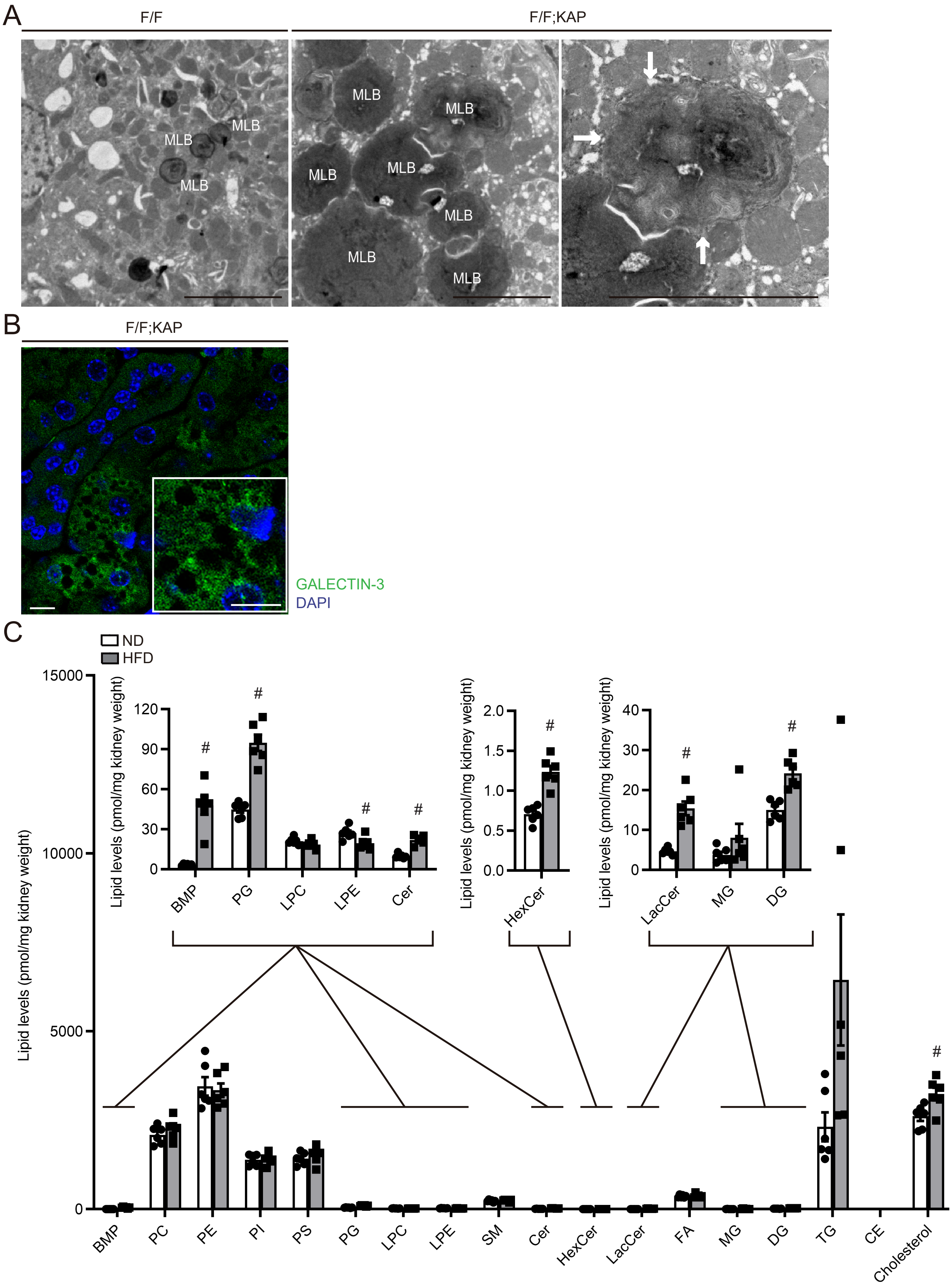
A
F/F
F/F;KAP
MLB
MLB
MLB
MLB
MLB
MLB
MLB
MLB
MLB
MLB
B
F/F;KAP
GALECTIN-3
DAPI
C
ND
HFD
Lipid levels (pmol/mg kidney weight)
15000
10000
5000
0
BMP
PC
PE
PI
PS
PG
LPC
LPE
SM
Cer
HexCer
LacCer
FA
MG
DG
TG
CE
Cholesterol
120
90
60
30
0
2.0
1.5
1.0
0.5
0.0
40
30
20
10
#

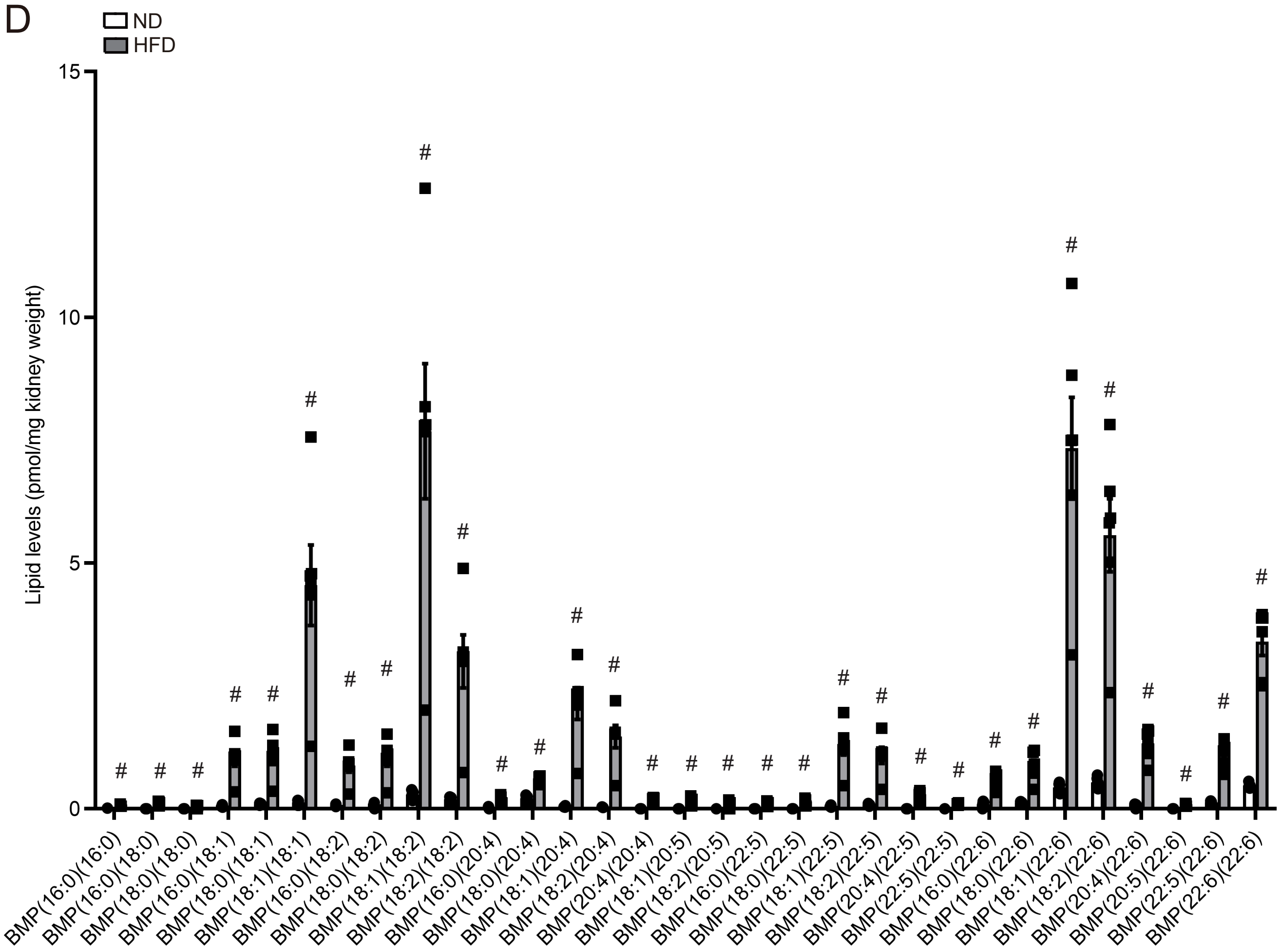

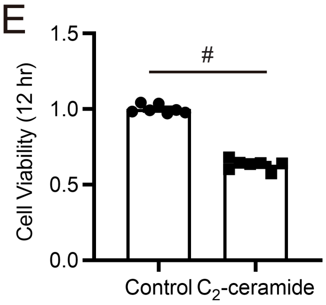

Supplemental Figure 7. Toxic lipids released from MLBs cause tubular injury after IR. (**A** and **B**) MLBs in PTECs of obese $Tfeb^{F/F}$;KAP mice are more susceptible to LMP. (**A**) Electron micrograph images of the kidneys of obese $Tfeb^{F/F}$ or $Tfeb^{F/F}$;KAP mice 2 days after unilateral IR operation. MLBs in PTECs of obese $Tfeb^{F/F}$ or $Tfeb^{F/F}$;KAP mice display interruptions of their membrane. (**B**) Immunostaining images of GALECTIN-3 in kidney cortical regions of obese $Tfeb^{F/F}$;KAP mice 2 days after unilateral IR operation. Sections were counterstained with DAPI. (**C** and **D**) Lipidomics of lysosome-enriched fraction of nonobese and obese mice (n = 6). Data are presented as the amount of each phospholipid species normalized to kidney weight. Cholesterylester was not detected. (**E**) Cell survival of PTECs treated with 100 μM C_2-ceramide for 12 hours (n = 7). Bars: 5 μm (**A**) and 10 μm (**B**). Values represent means ± SE. Statistically significant differences: $^{\#}P < 0.05$ versus nonobese mice or versus vehicle-treated cells (**C-E**, two-tailed Student’ s *t*-test). All images are representative of multiple experiments. Arrows indicate interrupted lysosomal membrane. F/F, $Tfeb^{F/F}$ mice; F/F;KAP, $Tfeb^{F/F}$;KAP mice; MLB, multilamellar body; BMP, bis(monoacylglycerol) phosphate; PC, phosphatidylcholine; PE, phosphatidylethanolamine; PI, phosphatidylinositol; PS, phosphatidylserine; PG, phosphatidylglycerol; LPC, lysophosphatidylcholine; LPE, lysophosphatidylethanolamine; SM, sphingomyelin; Cer, ceramide; HexCer, hexose ceramide; LacCer, lactosylceramide; FA, fatty acid; MG, monoacylglycerol; DG, diacylglycerol; TG, triacylglycerol; CE, cholesterylester; Chol, cholesterol.

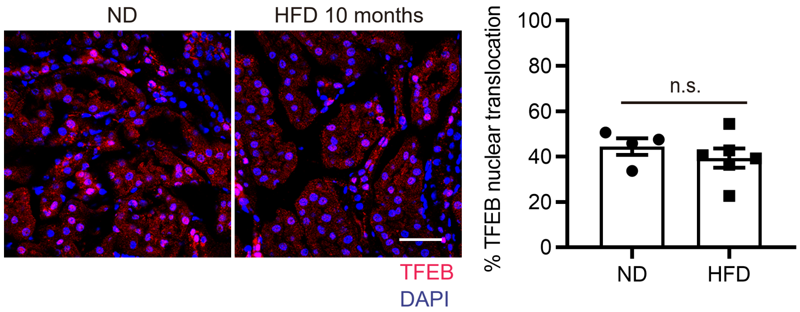

Supplemental Figure 8. Long-term lipid overload leads to TFEB downregulation. Representative immunostaining images for TFEB in the kidneys (n = 4–6). The percentage of PTECs exhibiting TFEB nuclear translocation was determined. Bars: 40 μm. Values represent means ± SE. Statistically significant differences: #*P* < 0.05 versus nonobese mice (two-tailed Student' s *t*-test). n.s., not significant.

Supplemental Table 1. ChIP-Atlas enrichment analysis using RNA-seq data from cultured PTECs after BSA or PA treatment. Transcription factors with enriched binding within 1 kb from transcription start sites were analyzed. An extensive comparison with publicly available datasets was conducted using the ChIP-Atlas (http://chip-atlas.org/). Top 10 rankings are shown.

ID	Antigen	Cell	Number of peaks	Overlaps /Our data	Overlaps/Control	Fold enrichment	$-Log_{10}(P)$	$-Log_{10}(Q)$
SRX3768263	USF2	K-562	1241	78/817	390/18046	4.41763	-24.4246	-20.5848
SRX150493	NFE2	K-562	2132	52/817	251/18046	4.57603	-16.9345	-13.9697
SRX150731	NFE2	GM12878	967	72/817	470/18046	3.38371	-16.8159	-13.9489
SRX3768264	USF2	K-562	947	45/817	188/18046	5.28705	-16.7418	-13.9232
SRX2250891	TFEB	HUVEC	1885	32/817	90/18046	7.85356	-16.0952	-13.3857
SRX150394	SREBF2	Hep G2	144	15/817	12/18046	27.6102	-13.4764	-11.1616
SRX7572083	SREBF1	KYSE-150	1160	43/817	223/18046	4.25915	-13.1494	-10.8836
SRX1131597	CBFA2T2	NCCIT	464	29/817	111/18046	5.77077	-11.8011	-9.65588
SRX573034	AFF4	HeLa	760	45/817	273/18046	3.6409	-11.6297	-9.50168
SRX7572085	SREBF1	TE-5	944	34/817	179/18046	4.19551	-10.3679	-8.36059

Supplemental Table 2. Patient characteristics. Kidney specimens were obtained from 146 patients. All continuous variables are expressed as median (interquartile range). Categorical variables are expressed as number (proportion). BMI, body mass index; eGFR, estimated glomerular filtration rate.

Subjects, n	146
Age (years)	47 (35–62)
Male sex, n (%)	65 (44.5)
BMI (kg/m^2)	24.3 (20.6–27.6)
eGFR (mL/min/1.73 m^2)	59.3 (46.5–82.5)
Urine protein/creatinine (g/gCre)	1.21 (0.43–3.29)

Supplemental Table 3. Characteristics of patients whose kidney sections were immunostained. Kidney specimens were obtained from 30 patients. All continuous variables are expressed as median (interquartile range). Categorical variables are expressed as number (proportion). BMI, body mass index; eGFR, estimated glomerular filtration rate.

Subjects, n	30
Age (years)	53 (44–64)
Male sex, n (%)	15 (50)
BMI (kg/m^2)	26.8 (22.1–29.2)
eGFR (mL/min/1.73 m^2)	57.1 (45.1–62.3)
Urine protein/creatinine (g/gCre)	1.96 (1.18–3.33)

Supplemental Table 4. Fatty acid composition of the high-fat diet.

	Percentage of total fatty acids (%)
Lauric acid, C12:0	0.19
Myristic acid, C14:0	1.61
Pentadecanoic acid, C15:0	0.09
Palmitic acid, C16:0	24.27
Palmitoleic acid, C16:1	2.36
Stearic acid, C18:0	13.82
Oleic acid, C18:1	42.07
Linoleic acid, C18:2	12.11
α -linolenic acid, C18:3	0.85
Arachidic acid, C20:0	0.21
Eicosenoic acid, C20:1	0.67
Eicosapentaenoic acid, C20:5	0.00
Behenic acid, C22:0	0.02
Docosapentaenoic acid, C22:5	0.00
Docosahexaenoic acid, C22:6	0.00
Lignoceric acid, C24:0	0.01
Tetracosenoic acid, C24:1	0.00
Others	1.71
Saturated fatty acids	40.23
Monounsaturated fatty acids	45.11
Polyunsaturated fatty acids	12.96
